# Structural dissection of sequence recognition and catalytic mechanism of human LINE-1 endonuclease

**DOI:** 10.1093/nar/gkab826

**Published:** 2021-09-23

**Authors:** Ian Miller, Max Totrov, Lioubov Korotchkina, Denis N Kazyulkin, Andrei V Gudkov, Sergey Korolev

**Affiliations:** Edward A. Doisy Department of Biochemistry and Molecular Biology, Saint Louis University School of Medicine, St. Louis, MO 63104, USA; Molsoft LLC, San Diego, CA 92121, USA; Genome Protection, Inc., Buffalo, NY 14203, USA; Genome Protection, Inc., Buffalo, NY 14203, USA; Genome Protection, Inc., Buffalo, NY 14203, USA; Roswell Park Comprehensive Cancer Center, Buffalo, NY 14263, USA; Edward A. Doisy Department of Biochemistry and Molecular Biology, Saint Louis University School of Medicine, St. Louis, MO 63104, USA

## Abstract

Long interspersed nuclear element-1 (L1) is an autonomous non-LTR retrotransposon comprising ∼20% of the human genome. L1 self-propagation causes genomic instability and is strongly associated with aging, cancer and other diseases. The endonuclease domain of L1’s ORFp2 protein (L1-EN) initiates *de novo* L1 integration by nicking the consensus sequence 5′-TTTTT/AA-3′. In contrast, related nucleases including structurally conserved apurinic/apyrimidinic endonuclease 1 (APE1) are non-sequence specific. To investigate mechanisms underlying sequence recognition and catalysis by L1-EN, we solved crystal structures of L1-EN complexed with DNA substrates. This showed that conformational properties of the preferred sequence drive L1-EN’s sequence-specificity and catalysis. Unlike APE1, L1-EN does not bend the DNA helix, but rather causes ‘compression’ near the cleavage site. This provides multiple advantages for L1-EN’s role in retrotransposition including facilitating use of the nicked poly-T DNA strand as a primer for reverse transcription. We also observed two alternative conformations of the scissile bond phosphate, which allowed us to model distinct conformations for a nucleophilic attack and a transition state that are likely applicable to the entire family of nucleases. This work adds to our mechanistic understanding of L1-EN and related nucleases and should facilitate development of L1-EN inhibitors as potential anticancer and antiaging therapeutics.

## Graphical Abstract

**Graphical Abstract:**
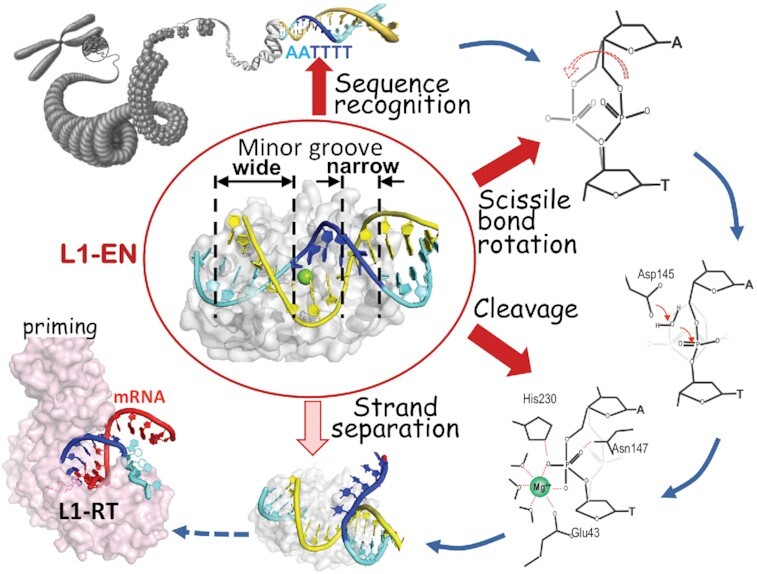
Mechanism of DNA sequence recognition and cleavage by L1-EN endonuclease domain during initiation of the LINE-1 retrotransposon target-primed reverse transcription.

## INTRODUCTION

The autonomous non-LTR retrotransposon long interspersed nuclear element-1 (LINE-1 or L1) is one the most abundant mobile elements in the human genome, comprising 17–20% of its content (1). While most L1 copies are defective (truncated, mutated or fragmented) and retrotranspositionally-incompetent, about 100–150 copies per genome can copy and paste themselves from one genomic location to another, contributing to increasing genomic instability throughout an individual's life. Moreover, even retrotranspositionally-incompetent L1 elements can cause genomic changes by creating DNA breaks, by supporting retrotransposition of non-autonomous short interspersed nuclear elements (SINEs) (2), and by facilitating ectopic or nonallelic homologous recombination (3). L1 elements have two open reading frames (ORF) encoding proteins (ORF1p and ORF2p) that bind the L1 mRNA to form ribonucleoprotein particles (RNP) as retrotransposition intermediates (4–12). ORF1p acts as a nucleic acid chaperone that binds L1 RNA (13–16), while ORF2p provides endonuclease (EN), reverse transcriptase (RT) and RNA-binding activities through three distinct functional domains (7,17–21). L1 retrotransposition proceeds via a target-primed reverse transcription mechanism (22,23). L1-EN initiates de novo L1 integration by producing a single-strand nick at a genomic site generally fitting the consensus target sequence 5′-TTTTT/AA-3′ (6,7,24,25). The nicked poly-T DNA strand with a free 3′ hydroxyl group is used as a primer for reverse transcription of the L1 RNA within the RNP (26,27), which is followed by second strand DNA synthesis and integration of the new L1 copy into the targeted genomic site.

Expression and/or new insertions of L1 can have various DNA damaging effects ranging from introduction of DNA breaks to oncogene activation. The process also impacts the immune system and is associated with multiple significant human diseases including metabolic, neurological and autoimmune disorders (28) and cancer (29,30). Hypomethylation of the intrinsic CG-rich promoter of L1 is a prognostic biomarker for many types of cancer (31) and ∼50% of all cancers have somatic integration of L1 (32). L1 activity also plays a role in age-related genomic instability, inflammation and pathologies such as neurodegeneration (33–39). Experiments in SIRT6-deficient mice, which display both elevated L1 activity and shortened lifespan, demonstrated that accumulation of L1 cDNA in the cytosol drives type I interferon production and that this response contributes to the cellular and physiological pathologies in the mutant animals (40). Furthermore, L1 expression and L1-mediated induction of interferon were found to be elevated in aged wild type mice while L1 inhibition led to reduced inflammation and a decrease in aging biomarkers (40–42). Overall, a large body of evidence suggests that inhibition of L1 activity is a promising strategy for development of novel therapeutics against aging, cancer, and other diseases. Non-specific strategies including downregulation of L1 expression through inhibition of demethylation of its promoter (43,44) or use of histone deacetylase inhibitors (45) and inhibition of RT activity using HIV-specific drugs nevirapine (NVR) and efavirenz demonstrated significant beneficial effects in cancer and aging model systems (40,46). However, development of effective L1-specific inhibitors requires an improved understanding of L1’s structural details and mechanism of action.

In particular, the endonuclease L1-EN is an attractive therapeutic target since its nicking activity alone can have a deleterious effect on genome stability and is essential for the following RT reaction, and thus, for all subsequent steps in L1 retrotransposition. L1-EN belongs to the family of metal-dependent phosphohydrolases that includes apurinic/apyrimidinic endonuclease 1 (APE1) and DNase I (21,47), which are part of the exonuclease-endonuclease-phosphatase (EEP) domain superfamily (48). A unique property of L1-EN and APE1-type domains of other non-LTR retrotransposons is their sequence-specificity (49). In fact, L1-EN is the major factor determining L1 integration target site specificity (23,27,32,50,51) fitting the defined consensus sequence motif, which has been confirmed both *in vitro* in cultured human cells (50) and *in vivo* in 2954 human tumor samples representing 38 different types of cancer (32). The mechanism(s) underlying L1-EN’s sequence-specificity is particularly intriguing since its closest structural homolog, APE1 (6,21,52), its paralog APE2, and other structurally related nucleases including DNaseI and Exo III do not target specific sequences. It was hypothesized that L1-EN may recognize specific conformation of DNA helices at polyA tracts due to a potentially narrowed minor groove and greater flexibility, particularly, at TpA steps (6,7,21). In turn, unlike APE1, L1-EN does not cleave DNA at abasic sites (6) and does not have exonuclease activity (24). These differences are puzzling from a structure-function perspective since crystal structures of the L1-EN apo-enzyme (PDB: 1VYB) showed that it shares the same structural fold as APE1 and that the two enzymes have highly conserved active sites (21,24,53). However, none of the previously reported L1-EN structures included a DNA substrate. Prior attempts to investigate the mechanism of L1-EN sequence specificity using computer modeling based on known structures of APE1 complexes with DNA, extensive L1-EN mutagenesis, and solution structures of polyA/polyT DNA (54,55) suggested an APE1-like nucleotide flipping-out recognition model (21,53). However, the possibility of an alternative model not involving nucleotide flipping-out was also considered (24). Overall, the existing data are not sufficient to conclusively identify a model that fully explains the structural basis of L1-EN’s sequence specificity and mechanism of the catalysis.

Here, we aimed to directly investigate the structural basis for the unique DNA sequence targeting capacity of L1-EN and its mechanism of catalysis by solving crystal structures of L1-EN complexed with two different DNA substrates containing the enzyme's preferred target sequence and the crystal structure of L1-EN with a coordinated Mg^2+^ ion. Two mutations were introduced into L1-EN to facilitate crystallization of the enzyme complexed with DNA: D145A to prevent DNA cleavage (6) and Y226K to alter crystal packing. The resulting data demonstrate that conformational properties of the preferred nucleotide sequence are key determinants of L1-EN’s capacity for sequence-specific DNA recognition. Remarkably, in one of the L1-EN–DNA complexes that we crystallized, the scissile bond phosphate adopts two alternative conformations, pointing to potential conformational changes occurring during catalysis. Computer modeling allowed us to propose a mechanism of catalysis that is likely shared by the entire family of nucleases. Our structural and modeling data suggest that conformational properties of the target DNA sequence drive both preferred binding and catalysis. Unlike related nucleases, which bend the DNA helix to aid in accessing the minor groove and potentially destabilizing the scissile bond, L1-EN compresses the DNA helix near the cleavage site. Such a mechanism potentially provides multiple advantages for the enzyme's specific task in L1 retrotransposition, including targeting genomic sites containing the poly-T sequence required for RT priming, promoting melting of the poly-T strand, keeping the targeted DNA site in the vicinity of L1 RNPs post-cleavage, and preventing nuclease activity during DNA synthesis.

The unique mechanistic features of L1-EN’s interaction with DNA revealed by this study will facilitate targeting of the L1 system to reduce its continuous DNA damaging effects that contribute to cancer, aging and other pathologies.

## MATERIALS AND METHODS

### Production of wild type and mutant LINE-1 endonuclease proteins

An N-terminal fragment of human LINE-1 encoding amino acids 1–238 of ORF2 (provided by Dr A. Osterman, Sanford Burnham Prebys Medical Discovery Institute, La Jolla, CA) was cloned into the pET28b+ based pSMT3 vector (provided by Dr R.A. Kovall, University of Cincinnati, Cincinnati, OH) using the Gibson Assembly protocol. The resulting plasmid, pSMT3-LINE-EN, directs expression of LINE-1 endonuclease (L1-EN) with an N-terminal 6xHis-SUMO tag for use in protein purification. pSMT3-LINE1-EN was transformed into BL21* *E. coli* cells (Invitrogen). Cell cultures were grown in Terrific Broth with shaking at 37°C to OD600 = 1.5. Protein expression was then induced by adding IPTG to 1 mM final concentration and incubating cells with shaking at 16°C overnight. The cell pellet was suspended in purification Buffer A (1 M NaCl, 25 mM HEPES pH 7.5, 10% glycerol, 1 mM Tris(2-carboxyethyl)phosphine hydrochloride (TCEP.HCl) containing 50 μg/ml leupeptin, 50 μg/ml aprotinin, 1 mM PMSF, 2 mM CHAPS and 0.1% Brij35. The cell suspension was frozen in liquid nitrogen and then cells were lysed by thawing followed by four cycles of sonication at 50% power, 50% pulsar setting for 3 min each. The lysate was clarified by centrifugation at 16,000 rpm for 50 min using a Sorvall RC5C centrifuge. The supernatant was mixed with 5 ml His60 NiNTA Superflow resin (TaKaRa) equilibrated with Buffer A. The solution was incubated on a rotator for 30 min at 4°C and then loaded onto a gravity column. After washing the resin with 300 ml of Buffer A containing 20mM imidazole, the protein was eluted with Buffer A containing 200 mM imidazole in two fractions of 10 ml each. The 6xHis-SUMO tag was cleaved from the protein with Ulp1 peptidase overnight at 4°C. The cleaved protein was diluted 5-fold with Buffer A without NaCl to a final concentration of 200 mM NaCl and applied to a 5 ml Hi-Trap heparin affinity column (GE Health Sciences). The protein was eluted with a gradient of NaCl (100 mM to 1 M) at ∼700 mM NaCl concentration.

Analytical ultracentrifugation (AUC) was used to measure potential protein oligomerization in solution. Purified protein was concentrated and extensively dialyzed against AUC buffer (25 mM HEPES pH 7.5, 100 mM NaCl, 1 mM TCEP). Sedimentation velocity studies were performed in a Beckman XL-A analytical ultracentrifuge at 20°C and 35 000 rpm. Absorbance at 280 nm was measured every 4 min for a total of 200 scans. The buffer viscosity and density as calculated by Sednterp (http://www.rasmb.org/sednterp) were 1.04913 ρ and 0.01436 η, respectively. These values were used to fit the data to the Lamm equation in SEDFIT software (56) using the continuous c(s) distribution model. Graphs were prepared using GUSSI software (UT Southwestern).

To produce D145A, Y226K and D145A/Y226K mutant forms of L1-EN, the desired mutations were introduced into the pSMT3-LINE1-EN construct using the QuikChange™ (Agilent) protocol adjusted to have partially-overlapping primers with 3′-overhangs (ThermoFisher Scientific). Mutations were confirmed by sequencing before transformation of constructs into BL21* cells. Mutant L1-EN proteins were expressed and purified as described above for WT L1-EN.

### Measurement of endonuclease activity

Endonuclease activity of WT and mutant L1-EN proteins was measured *in vitro* using a fluorescent DNA probe created by annealing two labeled oligonucleotides: 5′-/56-FAM/CCTTTTTTTTTAACCGC-3′ and 5′-GCGGTTAAAAAAAAAGG/3IABkFQ/-3′ (Integrated DNA Technologies) resulting in quenching of the FAM fluorescence signal. For each reaction, the indicated amount of recombinant L1-EN protein (50–400 ng) was incubated with 100 nM DNA probe in 20 mM HEPES pH 7.0, 50 mM NaCl, 1 mM MgCl_2_, 1 mM DTT, 0.1 mg/ml BSA, 10% glycerol and fluorescence was measured for 60 min at 37°C. In this assay, L1-EN-mediated nicking of the DNA probe results in separation of a fluorescein labeled product from the probe containing the quencher. The product was quantified by measuring fluorescence (TECAN Infinite M1000PRO, excitation wavelength: 495 nm (10 nm bandwidth); emission wavelength: 520 nm (10 nm bandwidth)). A linear range of the fluorescence change curve (typically 30 min) was used to calculate protein activity; results are expressed as relative fluorescent units (RFU) per min per ng protein and are shown as the mean ± standard deviation (SD) for 4–5 replicates, as indicated. Data were collected and averaged for several protein concentrations (19–90 nM for WT, 31–155 nM for D145A and 14–57 nM for Y226K) with duplicates for each concentration.

### Crystallization

**Figure 1.**
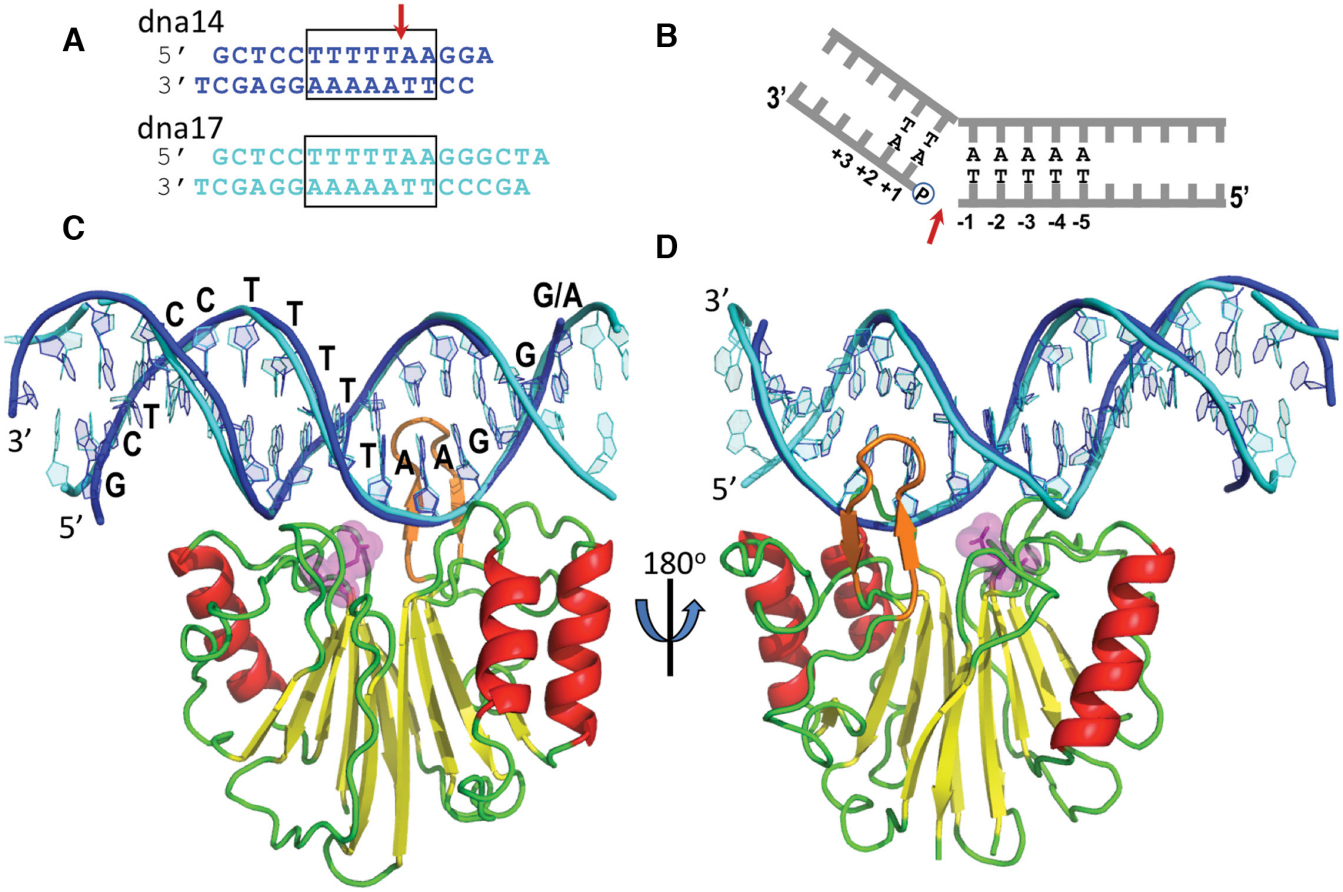
Structures of L1-EN complexes with dna17 and dna14 substrates. (**A**) Design of the two DNA substrates with the consensus target sequence boxed and the scissile bond marked by a red arrow. (**B**) Schematic representation of the preferred sequence-specific cleavage by L1-EN with numbering of base pairs relative to the scissile bond as used in the text. (**C**, **D**) Two views rotated by 180° of superimposed complexes of L1-EN with **dna17** (cyan) and with **dna14** (blue) in cartoon representation. Only one protein structure is shown using secondary structure-specific color-coding to simplify the view since protein structures in the two complexes are nearly identical. Glu43 is shown in stick and transparent magenta spheres representation. Loop βB5–βB6 is shown in orange.

WT and mutant L1-EN proteins were concentrated to 16–24 mg/ml in 300 mM NaCl, 15 mM HEPES pH 7.5, 2% glycerol, 1 mM TCEP. For co-crystallization with ***dna14*** and ***dna17*** DNA substrates (Integrated DNA Technologies, see Figure 1A), DNA was added to concentrated L1-EN in 1.2:1.0 DNA:protein ratio. Initial crystallization trials were conducted in 96–3 well sitting-drop plates (Intelli-Plate, Art Robbins Instruments) using a Phoenix robot with three different ratios of protein to buffer for each condition with commercial screens (Hampton Research, Molecular Dimensions). This was followed by designing new screens in 96-well format to refine crystallization conditions. The crystals reported here were obtained from the following conditions: (i) for crystallization of WT L1-EN: 0.1 M Tris acetate pH 6.0, 0.1 M lithium sulfate, 26% PEG 2000 MME; (ii) for co-crystallization of D145A/Y226K mutant L1-EN with ***dna17***: 0.1M MES pH 6.0, 0.2M NaCl, 30% Jeffamine ED-2003 from MIDASplus screen (Molecular Dimensions) and (iii) for co-crystallization of D145A/Y226K mutant L1-EN with ***dna14***: 0.1M sodium acetate pH 4.5, 0. 2M ammonium acetate, 15% PEG 4000 from ProPlex screen (Molecular Dimensions). Crystals were cryoprotected by adding 10% glycerol and 10% ethylene glycol and flash frozen either in liquid nitrogen or on a cold nitrogen gas stream. To assess Mg^2+^ ion coordination, crystals of WT L1-EN protein without DNA were soaked with 20 mM MgCl_2_ during cryoprotection for 30–60 s before freezing.

### Data collection and refinement

Data for the L1-EN D145A/Y226K-***dna14*** complex and for WT L1-EN coordinated with Mg^2+^ were collected using a home source X-ray generator Rigaku-MicorMax-007 HF with Raxis IV++ detectors and X-Stream nitrogen cooling system (Department of Biochemistry and Molecular Biology, Saint Louis University (SLU) School of Medicine, St. Louis, MO). Data for the L1-EN D145A/Y226K-***dna17*** complex were collected on the beam line 23-ID-B at the Advanced Photon Source, Argonne National Lab (Lemont, IL). Data were integrated and scaled using the HKL2000 program and structures were solved using the PHENIX software suite (57,58) with the molecular replacement method using coordinates of L1-EN (PDB ID: 1VYB) (21) as a search model. DNA substrates were manually built into electron density maps using the Coot program (59). Crystals of L1-EN with ***dna17*** belong to *P*4_3_2_1_2 space group with two complexes per asymmetric unit. DNA substrates were independently modeled in each complex into strong electron density sufficient to build all nucleotides and to unambiguously assign the majority of bases. Non-Crystallographic Symmetry averaging (NCS) was applied to protein molecules during model building and initial refinement cycles with torsion NCS restraints automatically defined in PHENIX. NCS restraints were omitted from the final refinement cycles. Crystals of L1-EN with ***dna14*** belong to *P*6_1_22 space group with one complex per asymmetric unit. Data collection and refinement statistics are presented in Table 1. DNA–protein interactions were analyzed and are presented using the DNAproDB online service (60). DNA conformation was analyzed using the 3DNA program (61).

**Table 1.** Data collection and refinement statistics

	L1-EN^a^ crystals formed with:		
Parameter	Mg^2+^	*dna17* DNA substrate	*dna14* DNA substrate
Wavelength (Å)	1.54	1.03	1.54
Resolution range (Å)	50–2.01	50.285–2.85	30.0–2.79
Space group	*P* 1 21 1	*P* 43 21 2	*P* 61 2 2
Unit cell dimensions			
*a*, *b*, *c* (Å)	42.86; 126.78; 44.85	100.77; 100.77; 140.77	91.61; 91.61; 229.82
α, β, γ (°)	90.00°; 98.94°; 90.00°	90.00°; 90.00°; 90.00°	90.00°; 90.00°; 120.00°
Total reflections	390 012	2 456 186	707 513
Unique reflections	30 980 (1505)^b^	17 800 (888)	14 939 (728)
Multiplicity	3.5 (3.0)	14.7 (14.8)	8.6 (9.0)
Completeness (%)	98.2 (93.7)	100 (100)	100 (99)
Mean *I*/ sigma (*I*)	16.3 (1.54)	40.5 (2.0)	18.5 (1.8)
Wilson *B*-factor	28.32	43.5	63.46
*R*-merge	0.089 (0.75)	0.063 (1.75)	0.139 (1.56)
*R*-pim	0.055	0.017 (0.464)	0.050 (0.548)
CC1/2	0.98 (0.627)	0.983 (0.759)	0.998 (0.675)
Refinement resolution range (Å)	33.3–2.01	39.19–2. 85	29.74–2.79
Reflections used in refinement	30 956 (1968)	17 438 (1615)	14 838 (1276)
Reflections used for *R*-free	1997 (142)	1744 (162)	1276 (142)
*R*-work	0.192 (0.267)	0.214 (0.333)	26.35 (40.58)
*R*-free	0.236 (0.309)	0.280 (0.415)	32.18 (48.58)
Number of non-hydrogen atoms	4045	5149	2527
Protein residues	465	464	232
Nucleotides		36	30
RMS (bonds)	0.008	0.008	0.006
RMS (angles)	1.099	1.263	0.818
Ramachandran favored (%)	98.5	96.30	93.04
Ramachandran allowed (%)	1.5	3.70	6.52
Ramachandran outliers (%)	0.0	0.0	0.43
Clashscore	5.23	12.36	14.95
Average *B*-factor	33.0	43.0	64.3

^a^Wild type L1-EN for crystals with Mg^2+^; L1-EN-D145A/Y226K for crystals with DNA substrates.

^b^Numbers in parentheses show corresponding values for high resolution shells. High resolution for crystals of WT L1-EN with Mg^2+^ are (2.04–2.01 Å) and (2.06–2.01 Å) for data collection and refinement statistics, respectively; (2.90–2.85 Å) and (2.93–2.85 Å) for mutant L1-EN complexed with ***dna17***; (2.85–2.80 Å) and (2.89–2.80 Å) for mutant L1-EN complexed with ***dna14***.

### Computer modeling of the DNA- and magnesium-bound L1-EN domain structure

We combined the obtained Mg^2+^ ion-bound L1-EN structure and ***dna14***-bound structures into a chimeric model containing the Mg^2+^ ion and DNA substrate including conformational changes associated with DNA binding. First, the chimeric model was built by ‘morphing’ the Mg^2+^ bound structure outside the immediate vicinity of the Mg^2+^ ion (12 Å) into the DNA-bound conformation. To accomplish this, harmonic tethers to the DNA-bound structure were applied and constrained force-field relaxation (ICMFF forcefield (62)) in internal coordinates was performed, while the active site region was constrained to the Mg^2+^ bound structure coordinates. Next, the resulting enzyme structure was combined with DNA in three alternative conformations: (i) the B-form conformation observed in all complexes, (ii) the alternative rotated conformation of DNA observed in the complex with ***dna14*** or (iii) an alternative model of the polynucleotide chain containing the -O-[PO_3_H]^–2^-O- (holophosphoric acid diester) link in the transition state based on the alternative rotated conformation. Trigonal bipyramidal geometry was derived from the small molecule crystallographic data found in the Crystallography Open Database (63,64) and kept fixed (other than bond torsion rotations). The DNA model was constrained to the B-form alternative of the DNA chain in the L1-EN complex structure. The ‘morphed’ Mg^2+^ bound enzyme and the polynucleotide chain were combined and relaxed together after removal of water molecules overlapping the DNA.

### Phosphate group rotation path modeling/animation

We modeled a putative path for the conformational transition of the scissile bond phosphate from the B-form conformation to an alternative conformation and a holophosphate intermediate/transition state. Torsion driving was performed, changing alpha and gamma angles of the phosphate in steps of 1/100 of the total change from the initial to final conformation. Other torsions were relaxed with constraints (harmonic constraints were applied to cartesian coordinates of atoms outside the phosphate link, and internal coordinates were also constrained harmonically to their values from the previous step to ensure smooth motion). All procedures were performed in ICM-Pro (Molsoft LLC, San Diego, CA) and implemented as scripts (available upon request).

## RESULTS AND DISCUSSION

### Determination of crystal structures for L1-EN complexed with DNA

We attempted to co-crystallize L1-EN with DNA using a mutant form of L1-EN in which a residue essential for catalysis, Asp 145, is mutated to alanine (D145A) to prevent DNA cleavage. A set of short double-stranded DNA substrates containing the target sequence TTTTTAA and single nucleotide T/A overhangs to facilitate crystal lattice formation was used for crystallization trials (Figure 1A). Several high-quality crystals diffracting to 2.0 Å resolution were obtained; however, all were found to consist of DNA-free protein. Analysis of protein contacts in crystal lattices in all published (PDB: 1VYB, 2V0S, 2V0R) (21,24,53) and newly obtained (our unpublished results) structures of L1-EN without DNA revealed a common protein–protein interaction interface, which includes the β-hairpin loop βB5-βB6 (amino acids 193–202) (Supplementary Figure S1). This loop is one of the major DNA contact areas in related nucleases including DNase I and APE1 and was proposed to similarly interact with the DNA minor groove in L1-EN (21,24,65,66). We hypothesized that involvement of this loop in protein–protein interactions of L1-EN (as observed in DNA-free crystals) might change its conformation in a way that prevents DNA binding. To disrupt this protein-protein interaction interface, we introduced a second mutation of Tyr226 to Lys (Y226K). L1-EN-Y226K mutant protein displayed full endonuclease activity (Supplementary Table S1). Higher activity of this mutant versus the wild type protein may be due to introduction of an additional positive charge next to the DNA binding site (10 Å away); however, additional experiments are required to test this hypothesis. Importantly, L1-EN-Y226K did not form crystals under previously established crystallization conditions. The double mutant protein L1-EN-D145A/Y226K was readily crystallized in complex with two DNA substrates, ***dna14*** and ***dna17***, and structures were solved to a resolution of 2.8 Å (Figure 1). There is one DNA-protein complex in an asymmetric unit in crystals of L1-EN-D145A/Y226K with ***dna14*** and two complexes in an asymmetric unit in crystals of the protein with ***dna17***. DNA was modeled into the electron density in each complex independently of other complexes to avoid a molecular replacement bias. These structures were deposited in the Worldwide Protein Data Bank (PDB) with accession numbers of PBD 7N8S for the L1-EN-D145A/Y226K/***dna14*** complex and PDB 7N94 for the L1-EN-D145A/Y226K/***dna17*** complex. L1-EN-D145A/Y226K will be referred to hereafter as simply L1-EN in the context of these protein–DNA complexes.

### DNA–protein interactions

In all three cases (two complexes of L1-EN with ***dna17*** and one with ***dna14***), the DNA substrate was bound to the protein in the same orientation and with similar distortions of the double helix conformation at the recognition site, as described below in more detail (Figure 1C, D; base pairs are numbered relative to the scissile bond position as indicated in Figure 1B). Notably, the preferred scissile bond (TTTT/AA) was juxtaposed with the catalytic site in all three complexes despite differences in the lengths of the two DNA substrates and in the contacts formed between the DNA and symmetry-related molecules in the crystal lattice (Supplementary Figure S2). The ends of the ***dna17*** substrate interact with symmetry-related proteins, resulting in unwinding of boundary base pairs. Crystal packing is completely different in the case of the L1-EN/***dna14*** complex, where the terminal single nucleotide overhangs form Watson–Crick base pairs with symmetry-related ***dna14*** molecules, thereby stabilizing the B-form helical conformation. Thus, the DNA helix is distorted at the boundaries of protein-bound ***dna17*** but is stabilized in protein-bound ***dna14***. Nevertheless, the conformation of the middle part of the DNA substrates containing the recognition sequence bound to the protein's active site are very similar in both ***dna14***- and ***dna17***-L1-EN complexes.

**Figure 2.**
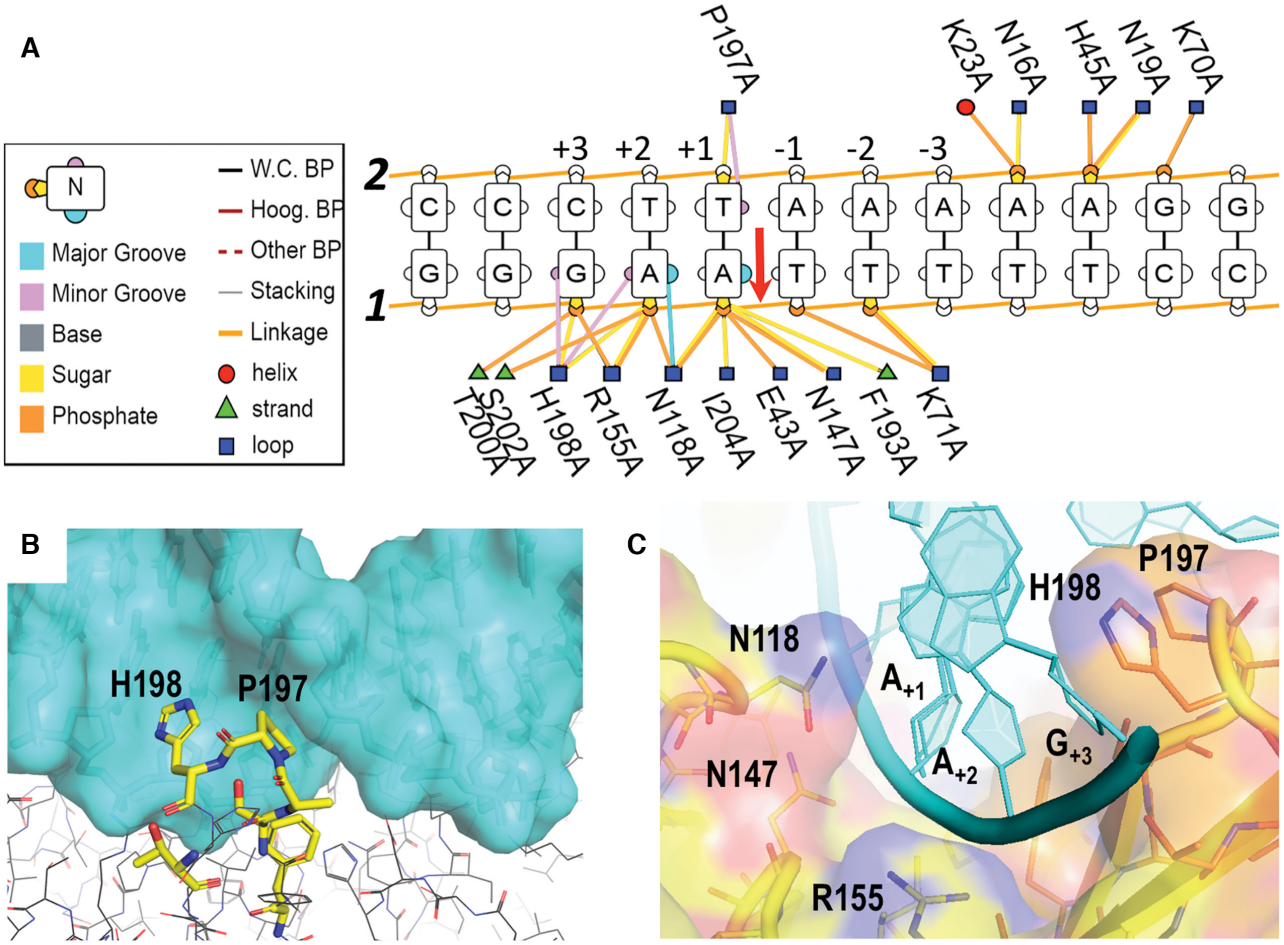
L1-EN interactions with DNA. (**A**) Scheme of DNA-protein interactions presented using the DNAproDB program. Symbols and colors depicting different elements of the DNA helix and protein secondary structure are shown in the boxed legend on the left. The scissile bond is marked by the red arrow. Strands 1 (cleaved) and 2 are designated by bold italic numbers. Base pair positions relatively to the cleavage site are numbered above strand 2. (**B**) Interactions of the βB5-βB6 β-hairpin loop shown in stick representation color-coded by atom type with the DNA minor groove shown in stick and transparent cyan surface representations. (**C**) Interaction of DNA strand 1 (cyan cartoon representation) with the cleft formed by two loops of L1-EN (βB5-βB6 and αB1-βB3) shown by transparent surface and stick representations color-coded by atom type.

These crystal structures show that L1-EN interacts with DNA almost exclusively through the sugar-phosphate backbone, with only a few interactions with bases (Figure 2A). The latter include insertion of Pro197 and His198 of the βB5-βB6 β-hairpin loop in the minor groove (Figure 2B) and interaction of Asn118 with the major groove. Insertion of this β-hairpin loop (βB5-βB6) into the minor groove is a prominent feature of DNA binding by related nucleases including DNase I (65) and APE1 (66), although the length and sequence of the loop varies between the different nucleases (21). Specifically, in L1-EN, the imidazole ring of His198 forms a polar contact with O4’ of G_+3_ and is 3 Å away from N3 of A_+2_. Pro197 forms Van der Waals contacts with T_+1_. The conformation of the βB5-βB6 loop in the DNA-bound complex differs from that in the apo structure, with the tip of His198 being 1.8 Å farther away from Asn118 in the αB1-βB3 loop (amino acids 117–120). The limited number of L1-EN interactions with bases in these structures rules out a conventional sequence recognition mechanism involving a network of nucleotide-specific hydrogen bonds. Base interactions by Pro197 and His198 of the βB5–βB6 β-hairpin loop and by Asn118 may contribute to recognition of two base pairs but cannot explain L1-EN’s preference for the entire target site. Consistent with this, previously published mutagenesis of this loop, including its replacement with loops from other retrotransposons did not result in altered sequence specific nicking (24). In this prior study, mutants with shortened loops were inactive, while that in which the loop was replaced by a longer loop of different amino acid sequence (LTx1, from a different retrotransposon) lost sequence specificity and ∼70% of the nicking activity of the wild type enzyme.

Most of the interactions between L1-EN and the ***dna14*** and ***dna17*** substrates are formed with phosphate groups of the nicked DNA strand (strand 1) near the scissile bond (nucleotide positions +3 to +1) (Figure 2A). The sugar-phosphate backbone of this strand is inserted into the cleft formed by the β-hairpin loop βB5–βB6 and the short loop αB1–βB3 (Figure 2C). This provides numerous polar and hydrophobic interactions that stabilize the conformation of the DNA strand with the scissile bond next to the L1-EN active site. OP1 of the scissile bond phosphate of A_+1_ is 3.1 Å from OE1 of Glu43, 2.9 Å from ND2 of Asn147 and 3.5 Å from the hydroxy group of Tyr115. Binding of the opposite DNA strand 2 is mediated by interactions between the phosphate groups at nucleotide positions -4 to -6 and an area of the protein with positive surface charge formed by Asn16, Asn19, Lys23, His45 and Lys70 (Figure 2A and Supplementary Figure S3). Both main interaction sites of strands 1 and 2 were previously identified by computer docking predictions (21,53) based on similarity with structures of APE1 and DNase-I complexes with DNA.

### Conformation of the DNA helix

**Figure 3.**
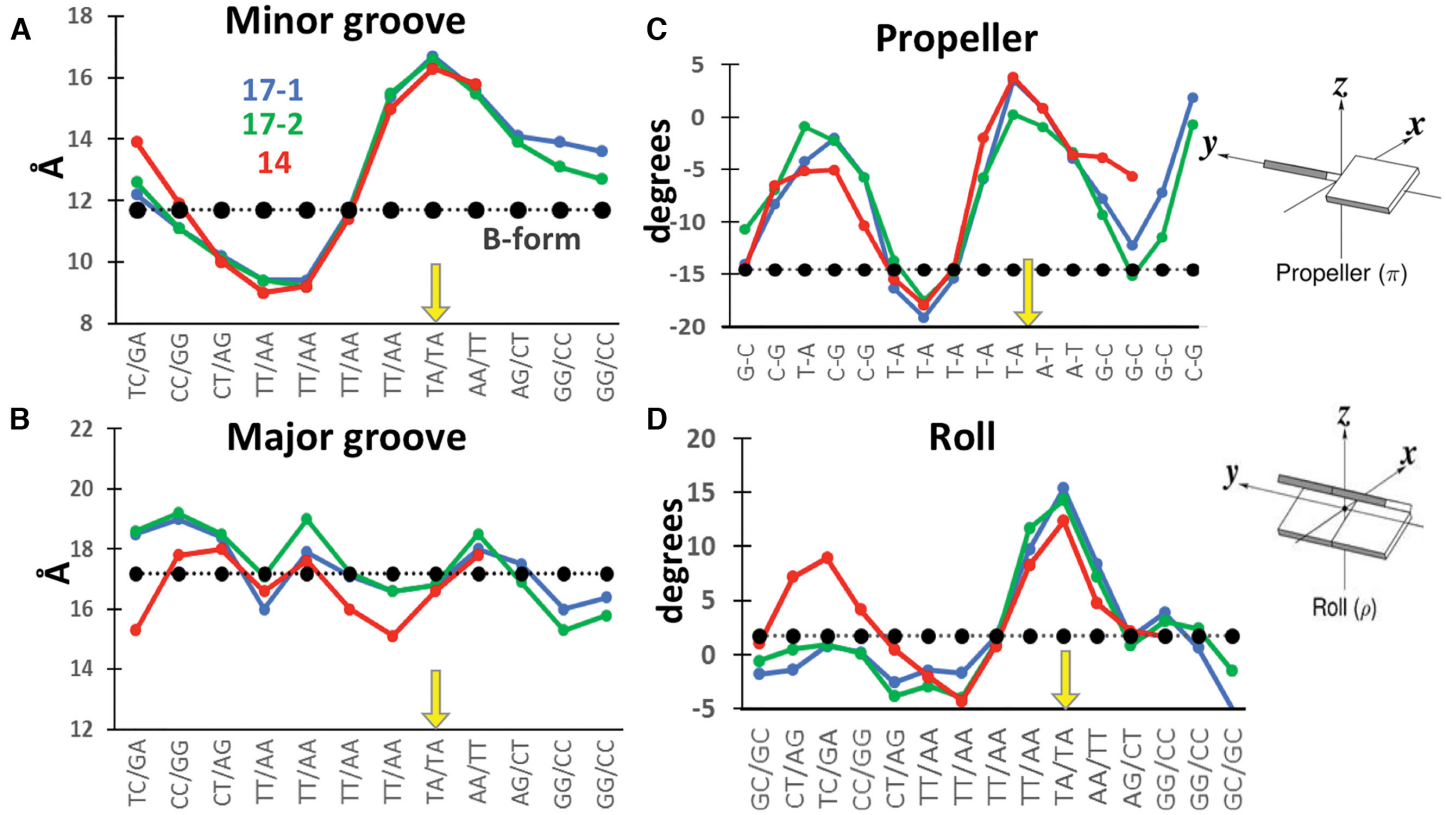
Geometry of DNA helices bound to L1-EN. Geometrical parameters of DNA substrates crystallized with L1-EN (two **dna17** shown in blue and green and **dna14** shown in red) are compared to canonical B-form DNA (shown in black) at each position of the DNA sequence shown on the x-axes. The position of the scissile bond is shown by the yellow arrow. (**A**) Minor groove width, (**B**) major groove width, (**C**) ‘propeller’ and (**D**) ‘roll’.

Our crystal structures show that the conformation of DNA bound to L1-EN deviates significantly from that of a canonical B-form helix. Remarkably, the observed deviations in geometrical parameters were almost identical in all complexes of L1-EN with DNA for the middle part of the DNA substrates, particularly base pairs in positions –4 to + 4 relative to the scissile bond (Figures 1 and 3). In contrast, conformations of the base pairs at the ends of the DNA fragments were found to differ between different crystal forms, reflecting unique interactions with symmetry-related L1-EN molecules in the crystals. These findings suggest that conformational changes around the cleavage site are stabilized solely by interactions of the DNA with L1-EN and are not affected by crystal packing. These data provide support for the sequence-specificity of DNA binding by L1-EN being largely defined by conformational properties of the DNA sequence.

Data for several geometrical parameters of the DNA helix calculated using the DNAproDB server that illustrate the conformational changes observed upon binding of DNA to L1-EN are shown in Figure 3. First, there is a significant widening of the minor groove at the cleavage site and downstream of the scissile bond (Figure 3A). The primary cause for this is the insertion of the βB5–βB6 β-hairpin loop into the minor groove (Figure 2B). Similar interactions of this characteristic loop were observed in APE1 (66,67) and DNase I (65,68) and were previously modeled for L1-EN (53). The side chain of His198 within the β-hairpin loop contacts nucleotides A_+2_ and G_+3_, pushing the latter away from the position that it would occupy in an ideal B-form helix and forcing widening of the minor groove to such an extent that its width becomes equal to that of a canonical major groove. The extensive contacts between the strand 1 sugar-phosphate backbone at positions +1 to +3 and the cleft formed by the βB5–βB6 and αB1–βB3 loops of L1-EN (Figure 2C) fix the position of this strand and contribute to conformational changes in the DNA. At the same time, the minor groove upstream of the scissile bond, at positions –4 and –5, is narrower in L1-EN-bound DNA than in standard B-form DNA (Figure 3A). This compression is likely due to interactions of strand 2 at these positions with the positively charged surface area of L1-EN formed by Lys23, His45 and Lys70 (Figure 2A, Supplementary Figure S3). In contrast to the minor groove, there is no significant deviation of the major groove width upon L1-EN binding (Figure 3B). Interestingly, the DNA helix is not bent significantly; this contrasts with the bending observed in complexes of DNA with DNAse I and APE1, as discussed further below (Figure 6). Deviations of polyA tract DNA geometry from canonical B-form was previously reported for free DNA in solution (55). In this study with a dT4A4 substrate (PDB:1RVI), the minor groove was widened by 2.3 Å at the TpA step and narrowed by 0.6 Å on the 3′ side relative to that in B-form. We found that similar deformations are significantly larger in DNA bound to L1-EN, with minor groove widening by 4.9 Å and narrowing by 2.5 Å at the corresponding positions. This suggests that polyT/polyA sequences represent a preferred binding sequence for L1-EN and that interaction of the protein with the sequence imposes additional conformational distortion on the DNA helix.

Substantial deviations in base pair geometry from B-form values are also similar in all obtained structures of L1-EN/DNA complexes. For example, there are significant rotations of bases relative to the opposite base (‘propeller’, Figure 3C) and relative to the base pair plane (‘roll’, Figure 3D). The largest changes in these parameters were found exactly at the scissile bond (yellow arrow in Figure 3C, D). These observations support the hypothesis of DNA conformation-driven sequence recognition by L1-EN (24).

Widening of the minor groove at the cleavage site and bending of the DNA helix are generally required for single-metal nucleases to access the minor groove (69). This can lead to weak preferences towards cleavage of sequences with greater conformational flexibility, as in the case of DNase I (65,70). Our data show that DNA bound to L1-EN fits this paradigm in terms of minor groove widening; however, there is no significant bending of the DNA helix (see Figure 6C). Rather, we observed compression of the minor groove upstream of the cleavage site which leads to distortion of base pair geometry and critical movement of the phosphate group, described below. This unique feature provides a plausible explanation for L1-EN’s strong preference for the extended sequence TTTTTAA, which is atypical of this class of nucleases, but has been well-documented through biochemical assays and analysis of *in vivo* integration sites (24). AT-rich sequences require less energy to adopt these conformational changes while T–A/A–T boundaries in DNA (as present in the L1-EN-targeted scissile bond) are characterized by minimal stacking interactions as compared to other combinations of base pairs (27,55). Therefore, the preferred sequence, TTTTTAA, allows widening and narrowing of the minor groove on either side from the scissile bond accompanied by rotation of base pairs more readily than other sequences. The structures of L1-EN DNA complexes reported here suggest that the established preferred cleavage site is also a preferred binding site, and thus, both binding and cleavage contribute to the overall sequence specificity of the enzyme's action. The requirement for a poly-T stretch instead of a mixed TA sequence is further dictated by the need for the nicked strand to serve as a primer for the RT reaction.

### Flexibility of the 5′ phosphate group of the scissile bond

**Figure 4.**
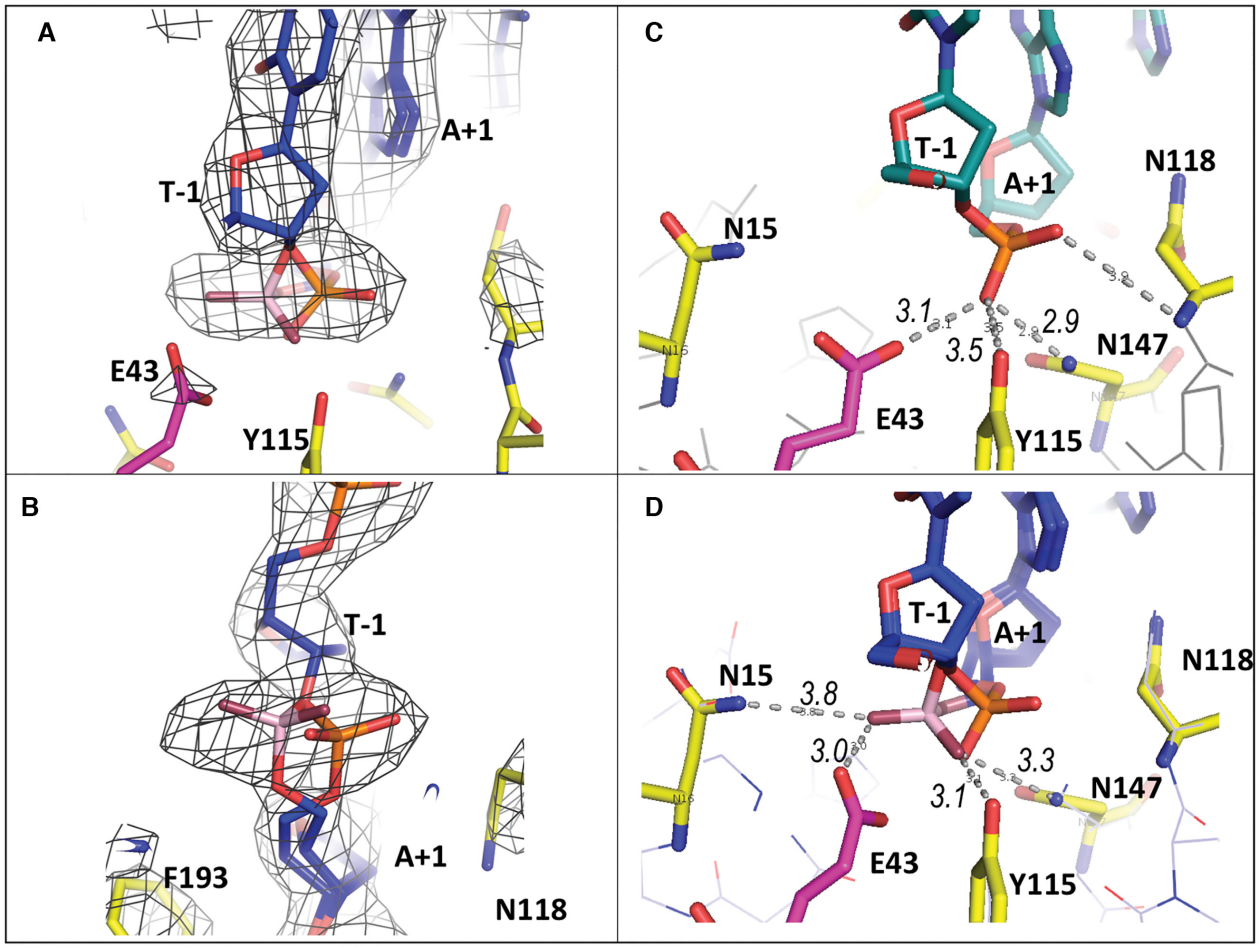
Alternative conformations of the scissile bond phosphate in the L1-EN-bound dna14 substrate. (**A**, **B**) Two different views of the 2*F*_o_ – *F*_c_ electron density map at 2σ level calculated using structure with omitted base pairs at T_-1_ and A_+1_ positions of the **dna14** substrate bound to L1-EN with two alternative conformations of the phosphate group. The phosphate in B form is shown in orange and red, while the alternative rotated conformation is shown in pink and red. Carbons of DNA are shown in blue. (**C**) Distances between the phosphate group of A_+1_ of **dna17** and active site residues of L1-EN. (**D**) Two conformations of the phosphate in **dna14** (colored as in A, B) with distances between the phosphate in rotated conformation and active site residues shown.

In the complex of ***dna14*** with L1-EN, we observed extended electron density around the A_+1_ phosphate at the scissile bond (Figure 4A, B). Electron density for the entire ***dna14*** backbone (especially its middle part) was well defined and the significant extension around the scissile bond phosphate cannot be attributed to low-resolution noise. Moreover, water or other solvent molecules cannot be fitted and refined in the extra density. Instead, we modeled two alternative conformations for this phosphate group with one corresponding to the typical conformation expected for a B-form helix (as observed in both complexes with ***dna17***, Figure 4C) and another where coordinated rotation of torsion bonds moves the phosphate closer to the active site residues, while largely preserving the positions of both 3′ and 5′ deoxyriboses (Figure 4D). The geometry of the alternative conformation of the phosphate group in ***dna14*** is not unprecedented. It is found in several structures in a previously compiled database of high-resolution DNA X-ray structures (e.g. DNA oligonucleotide, PDB:431D) (71,72) (Supplementary Table S2). The phosphate flip that we observed primarily involves large, coordinated changes in alpha and gamma torsion angles. Previous molecular dynamics simulation studies found that alpha/gamma transitions are improbable in free DNA, but are found in protein/DNA complexes (73).

The modeled rotation represents a less drastic conformational change than the previously described flipping out of a sugar-phosphate group in abasic DNA bound to APE1 (66,67). It is reasonable to hypothesize that the observed rotation of the phosphate group at the scissile bond is a consequence of conformational changes imposed on the DNA by protein interactions, particularly, of the minor groove compression described above combined with weak stacking and hydrogen bond interactions of bases in this part of the DNA. Rotation of the phosphate at the TA/AT transition was observed only in crystallized DNA (72), where the DNA was also significantly more deformed due to extensive crystal packing contacts compared to DNA in solution (55). Importantly, this rotation brings the scissile bond phosphate group closer to active site amino acids Glu43 and Asp145. Glu43 coordinates the required Mg^2+^ ion ((24) and below) and Asp145 analogs have been proposed to activate nucleophilic water in this nuclease class (21,66). Thus, the observed rotation brings the scissile phosphate into a position favorable for nucleophilic attack and the proposed transition state (see details below).

### Coordination of Mg^2+^ ion in the DNA-free crystal structure of L1-EN

**Figure 5.**
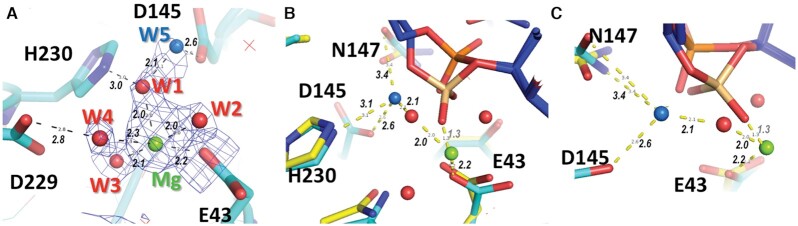
Coordination of Mg^2+^ ion in crystals of WT L1-EN soaked in MgCl_2_. (**A**) Mg-water cluster modeled into 2Fo-Fc electron density shown at 1.5 σ level calculated with omitted solvent. The Mg^2+^ ion is shown as a green sphere and water molecules coordinated by Mg^2+^ and by Asp145 are shown as red and blue spheres, respectively. Hydrogen bonds are shown by dotted lines with corresponding distances in Å. (**B**, **C**) Two views of superimposed structures of Mg^2+^-bound L1-EN (carbons shown in cyan) and L1-EN–**dna14** complex (carbons shown in yellow) with two conformations of the scissile bond phosphate group colored as in Figure 4. (B) View from the position of Mg^2+^ and Glu43. (C) View from the site of water molecule W5, coordinated by Asp145. In (B) and (C), the distance of 1.3 Å (grey number) between the Mg^2+^ ion and the phosphate in the rotated conformation was calculated using superimposed structures.

L1-EN, like other metal-dependent phosphohydrolases such as DNase I and APE1, uses a single divalent metal ion for catalysis (21,65). Coordination of a Mn^2+^ ion by the L1-EN active site residue Glu43 was demonstrated in a crystal structure of L1-EN with a mutated β-hairpin loop (PDB: 2V0S) (24). Similar positioning was observed for a Mg^2+^ ion in a DNase I structure (74), where it is coordinated by the corresponding Glu39 residue, and in APE1 structures, where it interacts with Glu96 (66,67,75), although a second metal ion was reported in another APE1 structure as well (PDB: 1E9N) (76). To confirm the position of the catalytic metal in L1-EN, we soaked crystals of WT L1-EN (formed without DNA) with 20 mM MgCl_2_ during cryoprotection and refined the structure to 2.0 Å resolution. The structure of Mg-bound L1-EN that we obtained (PDB 7N8K) was almost identical to the published L1-EN structure (PDB: 1VYB) (21), with a root-mean-square deviation (RMSD) of 0.1 Å, and to our DNA-bound L1-EN-D145A/Y226K structures. In the latter case, the RMSD was slightly higher, 0.4 Å, due to minor changes in the conformations of loop regions involved in the DNA binding interface. We modeled a Mg-water cluster with four water molecules at the electron density near Glu43 (Figure 5A). The refined position of the Mg^2+^ ion in our structure differs from that of Mn^2+^ in the 2V0S structure (24) by just 0.5 Å and is similar to the position of Mg^2+^ near Glu96 in APE1 (66,67,75) and Glu39 in DNase I (74). One of the Mg-bound water molecules is coordinated by Asp229 and one by His230. In addition to the four water molecules coordinated by Mg^2+^, there were other water molecules near the active site. The one with the strongest electron density is coordinated by Asp145 (2.6 Å) and Asn147 (2.7 Å). This water molecule coordinated by Asp145 and Asn147 is also present in previously published structures of L1-EN (1VYB) (21). Notably, Asp145 and corresponding aspartic acids in DNase I and APE1 were proposed to activate nucleophilic water during catalysis (21,66). Upon superposition of our Mg-bound L1-EN structure with the structures of our DNA-bound L1-EN complexes, distances between the modeled Mg^2+^ and the scissile bond OP2 are 3.8 Å for B-form DNA and 1.4 Å for the alternative conformation with the phosphate rotated as described above (Figure 5B, C). Modeling studies described below suggest that the intermediate holo-phosphate moiety at this position will be coordinated by the Mg^2+^ ion via two of its oxygen atoms.

### Comparison of L1-EN–DNA and APE1–DNA complexes

**Figure 6.**
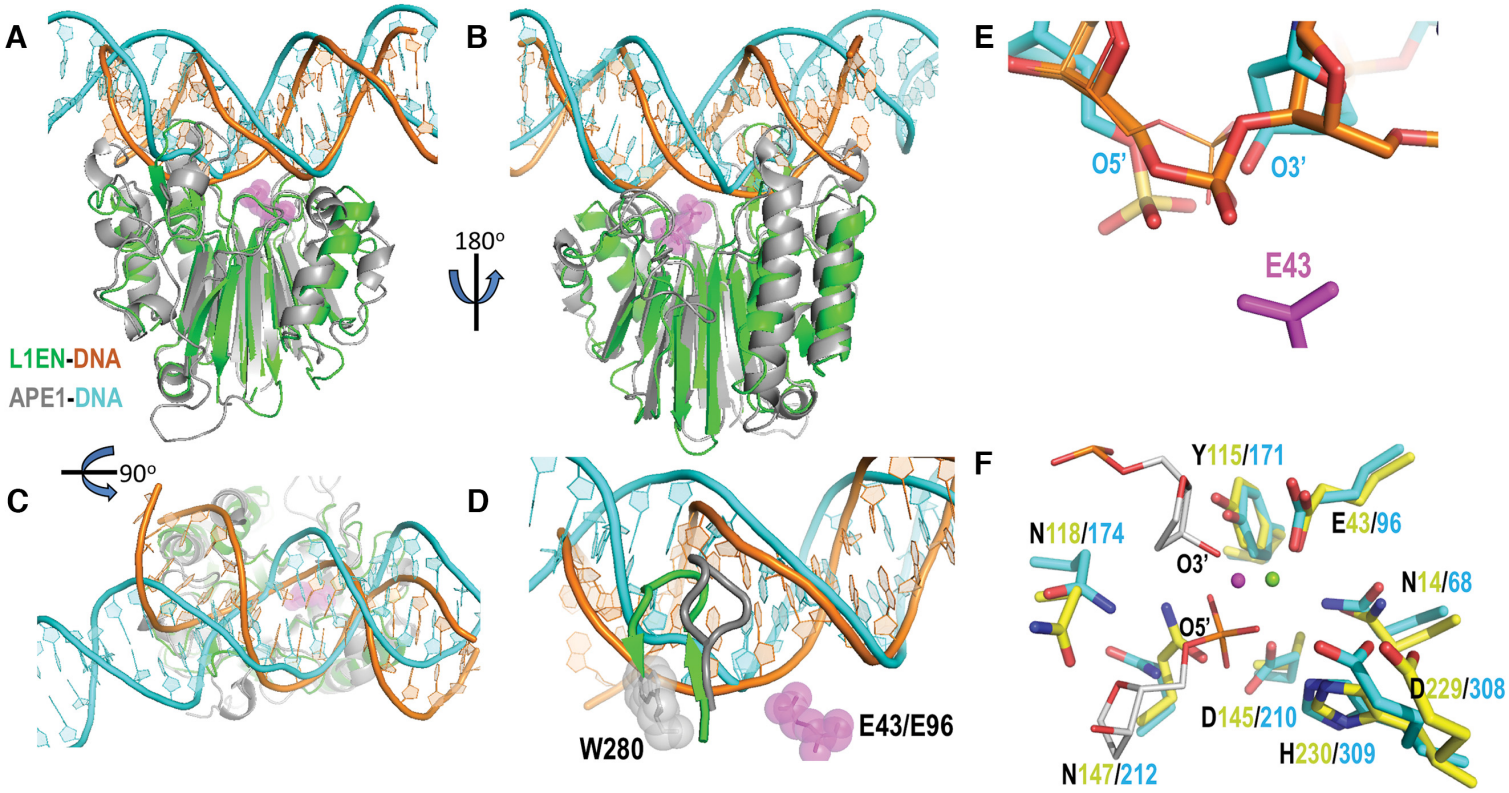
Comparison of L1-EN and APE1 structures. (**A**) Cartoon representations of L1-EN (green) bound to DNA (orange) and APE1 (grey) bound to DNA (5DFI, cyan). L1-EN is superimposed with APE1 by all Cα atoms. The catalytic E43 residue of L1-EN is shown by magenta sticks and spheres. (**B**) The same superimposed representations from (A) are shown rotated by 180° around the vertical axis. (**C**) The same superimposed representations from (A) are shown rotated by 90° around the horizontal axis (i.e. viewed from the top). (**D**) β-hairpin loops of L1-EN and APE1 with bound DNA are shown with color coding as in (A). Grey sphere representation: W280 of APE1; magenta sphere representation: E43 of L1-EN and E96 of APE1. (**E**) Representation of the scissile bond phosphate of DNA bound to L1-EN (orange and red sticks representation) with the phosphate in a rotated conformation superimposed with cleaved DNA product, corresponding to the cleaved DNA conformation, bound to APE1 (5DFF) (cyan, red and yellow). The B-form conformation of **dna14** bound to L1-EN is shown by thin lines. Complexes are superimposed by protein structures. Only E43 of L1-EN is shown for clarity. (**F**) Stick representation of active site amino acids of L1-EN (carbons shown in yellow) and APE1 (carbons shown in cyan) with corresponding numbers for each protein. The backbone of the DNA product bound to APE1 (5DFF) is shown by thin lines in grey, red and orange. Mg^2+^ is shown by a green sphere. Position of the 5′ phosphate of **dna14** is shown by a magenta sphere. Alanine is shown in the position of L1-EN D145.

APE1 is the closest structural homolog of L1-EN among crystallized EEP enzymes; the two proteins have the same secondary structure elements forming similar folds, with differences primarily in loop regions (21) (Figure 6 A-B). The catalytic sites of the two enzymes are highly conserved as well, with identical amino acids coordinating the scissile bond phosphate, catalytic metal ion and water molecules (Figure 6F). Comparison of our DNA-bound L1-EN structure with that of APE1 (5DFI) (67) showed that DNA duplexes interact with the two proteins through similar surface areas (Figure 6A, B). Notably, however, while the parts of the DNA helices upstream of the cleavage sites have similar orientations in both complexes, the downstream segment of DNA is significantly bent in APE1 complexes but not in L1-EN complexes (Figure 6C). Differences in the conformation of the downstream segment of DNA appear to be determined by differences in the length and amino acid compositions of the β-hairpin loop inserted in the minor groove of the DNA substrate and the loop that forms the opposite side of the cleft that accommodates the DNA (αB1–βB3 in the case of L1-EN). For both proteins, DNA inserted in the cavity between these loops forms multiple contacts stabilizing the conformation of the cleaved strand immediately downstream of the scissile bond (Figure 2C and (67)). In L1-EN, the DNA strand that will be nicked sits deep in this cavity with the phosphate group of the scissile bond situated only 3 Å away from the active site Glu43 residue (Figure 4C). APE1 has a less accommodating cavity than L1-EN due to the presence of Trp280 at the bottom of the cavity (versus Ser202 in L1-EN (Figure 6D)). Trp280 pushes the DNA helix away from the APE1 active site, requiring the abasic DNA backbone to form a sharp kink around the residue in order to approach the active site. This bending of the DNA helix caused by the conformational clash with Trp280 likely leads to the initial ‘flipping out’ of a deoxyribose into the active site (Figure 6D). The flipped-out conformation is then further stabilized by Arg177 occupying the position of the missing base. Ser202 of L1-EN permits the DNA strand to penetrate deeper into the cavity and approach the active site without flipping-out of a nucleotide. In the alternative conformation of ***dna14*** bound to L1-EN, the rotated-out phosphate group is even closer to Glu43 (Figure 4D). Superimposition of the L1-EN and APE1 protein structures determined from L1-EN-***dna14*** and APE1-DNA product (PDB:5DFF) complexes showed that the phosphate group of the scissile bond in the rotated conformation is located exactly between the 5′ and 3′ ends of the cleaved product DNA (Figure 6E), suggesting that the rotated out phosphate is close to a transition state conformation.

As mentioned above, conservation of the L1-EN and APE1 catalytic sites includes the amino acids that coordinate the scissile bond phosphate, catalytic metal ion and water molecules (Figure 6F). In L1-EN, metal ion-coordinating Glu43, water-coordinating His230 and Asn14, and 3′ deoxyribose-interacting Tyr115 adopt almost identical conformations to the corresponding Glu96, His210, Asn68 and Tyr171 residues in APE1. Side chains of amino acids Asn118, Asn147 and Asp229 coordinating water molecules in L1-EN are within 2 Å of their APE1 counterparts Asn174, Asn212 and His308. While Asp145 is mutated to alanine in our L1-EN-DNA complex, its Cα and Cβ atoms overlap with those of APE1 Asp210, which is proposed as a general base for activation of nucleophilic water. Taken together, these similarities suggest that L1-EN and APE1 should share the same catalytic mechanism even though positioning of the scissile bond phosphate at the active site is achieved through different conformational changes of DNA. Large conformational changes, such as the flipping-out of ribose and phosphate in DNA targeted by APE1, are not required for catalysis by L1-EN. The proximity of the DNA helix to the L1-EN active site explains the results of previously published mutagenesis used to test a hypothetical flip-out mechanism for L1-EN (24). Interaction of Ser202 with the phosphate at position +2 reflects the ∼70% loss of nicking activity seen with the S202A mutant. The observed inactivity of the I204Y mutant likely reflects hydrophobic interaction of Ile204 with the DNA backbone. The tyrosine hydroxyl would be expected to interfere with the backbone immediately downstream of the cleavage site. The R155A mutant, which showed 12% of wild type nicking activity, lacks two hydrogen bonds between Arg155 and phosphate groups at base pair positions +2 and +3. The absent/reduced activity of these mutants, which both alter the conformation of the nicked strand downstream of the cleavage site, confirms the importance of proper coordination of this part of the nicked DNA strand for catalysis.

### Modeling of the catalytic mechanism of L1-EN

**Figure 7.**
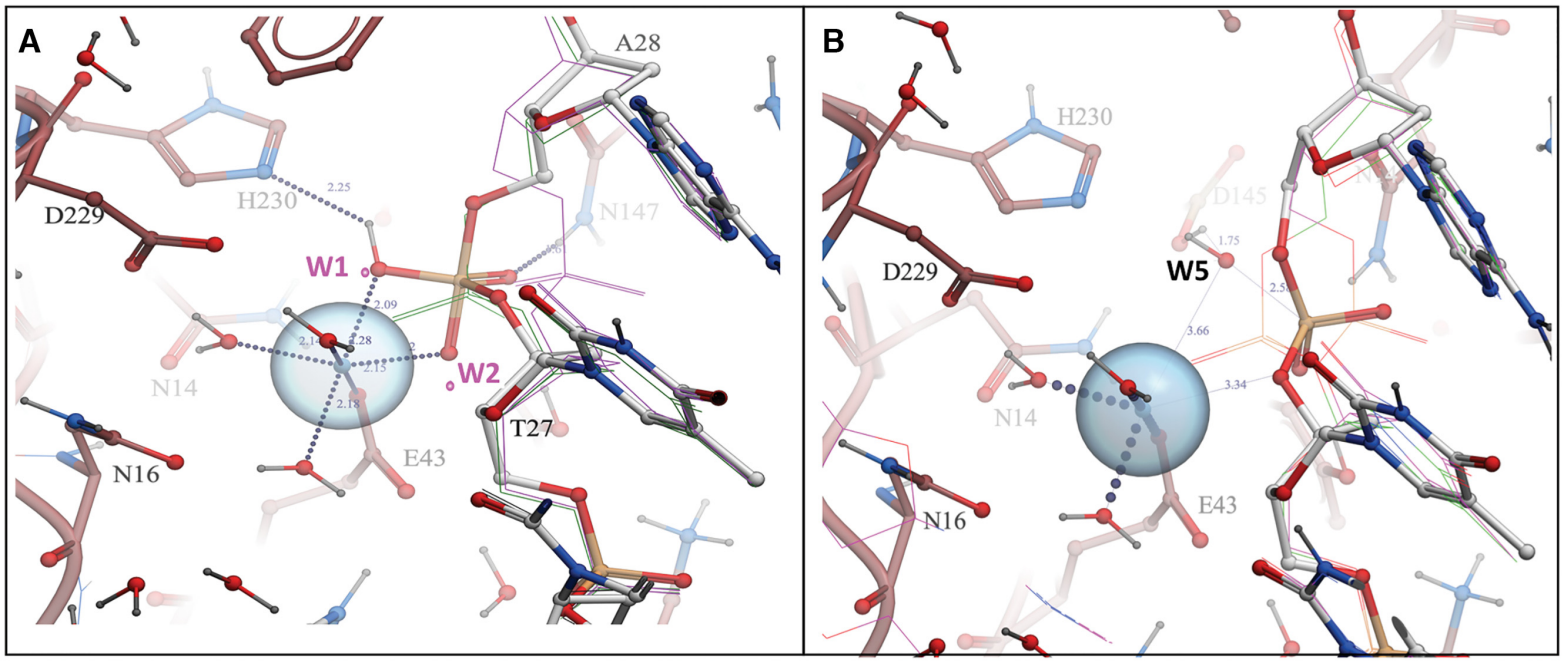
Model of transition states during L1-EN catalysis. (**A**) Transition state of the holo-phosphate group (shown by orange and red stick representation) interacting with octahedrally-coordinated Mg^+2^. Magenta dots indicate two water molecules (W1 and W2) coordinating the Mg^2+^ ion (transparent blue sphere) in the Mg-bound enzyme structure that are displaced by the holo-phosphate transition state moiety. (**B**) The phosphate groupat the intermediate position between the two conformations observed in our crystal structures (B-form vs rotated, shown by thin lines) is located near the crystallography-defined water molecule W5 coordinated by Asp145.

Proximity of the scissile bond to the active site in L1-EN–DNA complexes (Figure 5B) and the position of the rotated-out phosphate group between the two ends of the cleaved DNA product in the APE1–DNA complex (Figure 6E) suggest that this conformation corresponds to a transition state of the DNA backbone during cleavage. We took advantage of these data to model the mechanism of the L1-EN catalytic reaction with minimal conformation perturbations and assumptions. We modeled a L1-EN-DNA complex with a bound Mg^2+^ ion by forcefield relaxation in the mixed constraints (see Materials and Methods) with active site amino acids around Mg^2+^ coming from our structure of Mg^2+^-bound WT L1-EN without DNA and the rest of the amino acids coming from our structure of L1-EN-D145A/Y226K complexed with ***dna14***. These two crystal structures are quite close in the intermediate layer of 10–14 Å from the Mg^2+^ ion, with RMSD = 0.67 Å for all heavy atoms. This allowed a smooth transition without any significant distortions such as clashes, deviations of phi/psi pairs from favorable regions of the Ramachandran plot or unfavorable omega angles. Covalent geometry, i.e., bond lengths and angles, was automatically preserved in torsion-only relaxation. When DNA was introduced into the model of the complex with the scissile bond phosphate in the conformation of B-form DNA, one of the water molecules solvating Mg^2+^ (W2, Figure 5A) clashed with the 3′ ribose ring (C3′ and C4′ at ∼2.1 Å). At the same time, oxygen atoms of the scissile bond phosphate remained too far away (>3.8 Å) to coordinate Mg^2+^ favorably. However, in the alternative rotated conformation, this phosphate is much closer to the Mg^2+^ ion (within 1.4 Å) and displaces two water molecules from the Mg^2+^ coordination shell (W1 and W2, Figures 5A and 7A). The resulting Mg^2+^ coordination is not optimal, having five coordinating atoms instead of the strongly preferred six atoms in an octahedral arrangement. The position of OP2 between the two displaced water molecules prompted us to model a holo-phosphate diester (i.e. penta-coordinated phosphorus form of phosphate -O-[PO_3_H]^–2^-O-) as a favorable transition state of the hydrolysis reaction in place of the regular ortho-phosphate (-O-[PO_2_]^–^-O-). Indeed, when modeled into the rotated phosphate DNA conformation, the transient holo-phosphate moiety became strongly coordinated by the Mg^2+^ ion via two of its oxygen atoms (at 2.0 Å and 2.1 Å from Mg^2+^), replacing the two water molecules and maintaining near-octahedral Mg^2+^ coordination (Figure 7A). Strong preference of the hydrated Mg^2+^ ion for an octahedral configuration is well established (77). We propose that this strong preference is key for L1-EN catalysis, allowing the enzyme to lower the energy of the PO_5_ transition state (TS) with respect to the typically much more stable PO_4_ which would only contribute one oxygen atom to the Mg^2+^ coordination shell (green ‘wire’ in Figure 7A) while displacing two water molecules. It is possible that this state becomes a local minimum intermediate on the reaction path or a low enough saddle point to allow quick progression of the hydrolysis reaction.

An important mechanistic question is how the proposed intermediate pentavalent holo-phosphate state is formed. Asp210 in APE1 was proposed to serve as a general base (66). The equivalent residue in L1-EN, Asp145, occupies a similar position and coordinates a water molecule in the Mg^2+^-bound structure. Based on our structures, we hypothesize that the scissile bond phosphate rotates due to conformational changes imposed by interactions with L1-EN. Attraction of the negatively charged phosphate to the positively charged Mg^2+^ ion may stabilize rotation of the scissile bond phosphate out of the initial B-form conformation. We modeled the possible path of such rotation between the two conformations modeled in our crystal structure by internal coordinate driving. Notably, about halfway through the rotation, the phosphate approaches W5, which is coordinated by Asp145 and favorably positioned for nucleophilic attack on the phosphate (Figure 7B). Asp145 carboxylate may serve as a general base that accepts a proton from W5, thus liberating the hydroxyl anion. p*K*_a_ estimation for Asp145 using PROPKA (78) indicated a strong upshift of p*K*_a_ = 6.5 for this side chain, in agreement with a general base function. Thus, we propose that the activated water nucleophilic attack occurs as the phosphate undergoes rotation. As the hydroxyl and phosphate converge, the resulting holo-phosphate proceeds to form a Mg^2+^ complex (see Supplementary Movie 1).

We do not have data on the reaction path beyond the transition (or intermediate) state, but we can speculate that from this point, phosphate O3′ may accept a proton, potentially, from an adjacent Mg^2+^-bound water and disengage from the phosphate. The structure of APE1 complexed with its cleavage product (67) indicates that the phosphate of the cleaved product may rotate away from O3′ oxygen. Similar rotation appears possible in L1-EN–DNA complexes and is consistent with L1-EN’s preference for breaking the O3′-P bond rather than the O5′-P bond (6).

Our results support a single metal catalytic mechanism initially proposed for APE1 (66) and later confirmed in more detailed analyses (67,79). Homology of L1-EN and APE1 active sites and the similar location of the scissile bond in complexes of DNA with the two enzymes suggest that the above described steps of the catalytic mechanism are shared by both enzymes and, potentially, by other nucleases as well. Our structural analysis of the L1-EN-***dna14*** complex revealed rotation of the scissile bond phosphate group leading to two distinct states for the nucleophilic attack and a subsequent transition state. Similar local rotation is possible in a flipped-out conformation of DNA bound to APE1. Indeed, differences comparable to the phosphate rotation in L1-EN-bound DNA exist between conformations of APE1-bound DNA substrate (PDB 1DE8, (66)) and DNA product (PDB 5DFF (67)).

**Figure 8.**
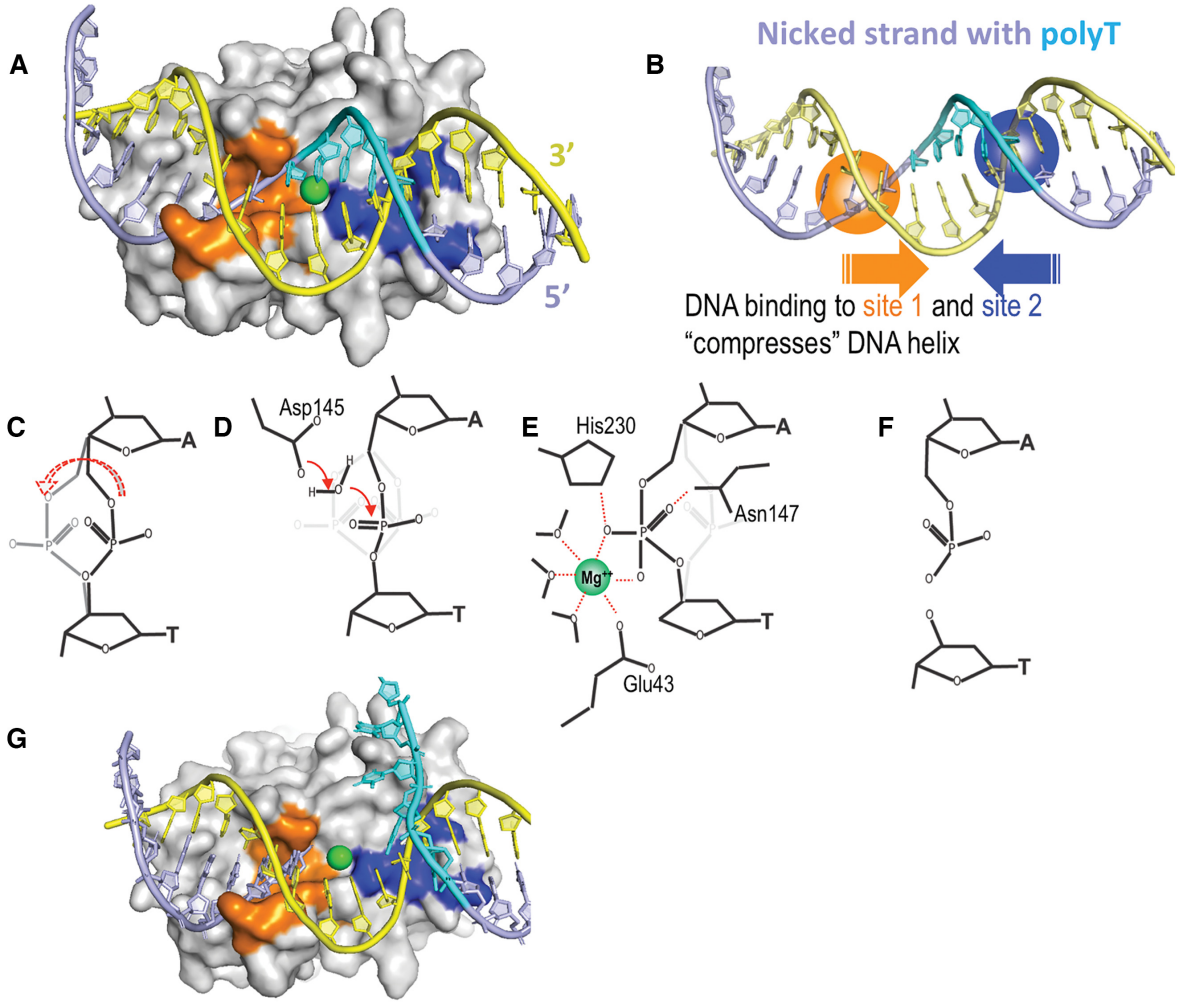
Proposed mechanism of sequence-specific cleavage by L1-EN. (**A**) L1-EN is shown in grey surface representation with two DNA-binding sites highlighted in orange (cleft binding nicked strand one at positions + 1 to + 3) and blue (positively charged area binding strand 2 at positions –4 to –6). Bound DNA is shown in cartoon representation with the nicked strand in light slate blue and its poly-T stretch in cyan. The opposite strand is shown in yellow. The green sphere (in A, E and G) represents the Mg^2+^ ion. (**B**) Schematic illustration of conformational pressure imposed by L1-EN on the DNA helix through interaction of site 1 (blue) with the nicked strand and interaction of site 2 (orange) with the opposite strand. (**C**) Rotation of the scissile bond phosphate group observed in the L1-EN–**dna14** crystal structure. (**D**) Model of the nucleophilic attack of the phosphate group by water activated by Asp145 during phosphate rotation. (**E**) Transition state of the holo-phosphate group interacting with octo-coordinated Mg^2+^. (**F**) Cleavage product. (**G**) Proposed step of dissociation of the nicked poly-T strand (cyan) from the L1-EN–DNA complex after cleavage due to the conformational distortion of the DNA described in (B), while the other DNA strands remain attached (representations and colors as in (A)).

Positioning of the scissile bond phosphate at the catalytic site is achieved by different mechanisms in these nucleases. In the APE1, it is achieved by a nucleotide flipping-out mechanism. The phosphate in the flipped-out conformation of DNA is positioned next to the catalytic residues Asp210 and Glu96. In the B-form conformation of DNA bound to L1-EN, the corresponding phosphate does not form bonds with nucleophilic water and the catalytic metal. These interactions occur only during rotation of the flexible phosphate group. In our L1-EN-DNA structures, there is no Mg^2+^ ion to attract negatively charged phosphate and stimulate its rotation. Therefore, we propose that this rotation is a result of the DNA helix distortion imposed by protein interactions leading to conformational tension around the scissile bond which forces the phosphate group out of the stable B-form conformation (Figure 8C). A mechanism based on compression-based distortion of the phosphate backbone is supported by L1-EN’s lack of exonuclease activity and inability to cleave single-stranded DNA (24) (even though single-stranded DNA can theoretically bind to the active site and would have greater conformational flexibility). Unlike APE1, L1-EN does not have exonuclease activity and at least two base pairs downstream of the cleavage site are required for efficient cleavage (24). Therefore, binding of the DNA helix up- and downstream of the cleavage site is critical for deformation of the DNA helix. This deformation does not lead to DNA bending, as in the case of APE1 and DNase I, but rather to compression of the DNA helix upstream of the cleavage site, as indicated by narrowing of the minor groove (Figure 3A). An unstable conformation of the scissile bond phosphate may decrease the energy barrier for phosphate activation by nucleophilic water (Figure 8D) and the lack of strong stacking interactions between AT and TA base pairs lowers the energetic barrier for such movement. Conformation-driven specificity provides a plausible explanation for the promiscuous nature of L1-EN’s ‘sequence-specific’ cleavage. Alternative target DNA sequences can be recognized and undergo similar conformational distortion but with much lower probability due to less stable DNA binding and higher energetic cost of scissile phosphate movement.

### Unique features of the L1-EN endonuclease mechanism and implications for retrotransposon activity

Evolution of L1-EN with a similar fold compared to related nucleases such as DNase I and APE1 presumably led to acquisition of novel properties required for its function within the context of L1 retrotransposition where it serves to provide a poly-T DNA strand as a primer for reverse transcription. How the poly-T strand dissociates from the L1-EN-bound nicked DNA and is transferred to the RT domain for priming has not been defined, although helicase-mediated unwinding was previously proposed (26). Our new structures of L1-EN-DNA complexes provide data supporting a unique mechanism of DNA binding that may serve two purposes: sequence-specific cleavage and DNA helix melting (Figure 8). Unlike DNase I and APE1, L1-EN does not significantly bend the DNA helix (Figure 6C). Instead, it compresses the helix between two binding sites as indicated by narrowing of the minor groove upstream of the scissile bond (Figures 3, 8B and Supplementary Figure S3). This feature is unique for L1-EN and may explain its requirement for a longer poly-T stretch for optimal binding. The compressed conformation is not relaxed after cleavage, and thus may lead to melting of the DNA helix in the poly-T region upstream of the cleavage site (Figure 8G). Unlike DNA bending at the cleavage site, which would not affect the conformation of the DNA helix upstream of the cleavage site, DNA compression between up- and downstream binding sites is expected to continue destabilizing Watson–Crick base pairing after cleavage. This would lower the energetic barrier for DNA helix melting, particularly at the poly-T stretch upstream of the nick, thus leading to its dissociation and availability as the primer required for reverse transcription (22,27). Alternatively, melted single-stranded DNA may serve as a substrate for initiation of helicase-mediated unwinding, a previously hypothesized mechanism for the primer generation (26).

Interactions of DNA with L1-EN downstream of the nick also seems to be fine-tuned for LINE-1 function. The multiple contacts of the DNA bound in the deep cleft between two loops of the enzyme (Figure 2C) suggest slow dissociation of the complex after nicking, which would aid in transfer of the melted poly-T strand to the L1-RT domain. This may also inhibit further endonuclease activity which would be counterproductive during the DNA synthesis step. The activity of L1-EN may be further regulated through protein-protein interactions between domains of ORF2p or even between different proteins in the oligomeric complex with ORF1p. For example, the protein interaction interface around Tyr226 found in all DNA-free L1-EN crystal structures may be involved in interdomain interactions within LINE-1 ORF2p or with ORF1p. L1-EN does exist in solution as a monomer at sub-millimolar concentrations (21) (Supplementary Figure S4); however, the presence and proximity of other proteins/domains may stimulate protein binding by this interface.

In conclusion, the structural and modeling studies reported here revealed a novel mechanism of sequence-specific binding and cleavage by L1-EN favorable for its activity within the context of L1 retrotransposition. This mechanism illustrates an elegant evolutionary solution for adaption of a common nuclease fold to specific functions. At the same time, our results provide new insights into the general mechanism of catalysis by related nucleases. Our findings regarding the positioning of DNA relative to the L1-EN active site and local conformational changes of the scissile bond phosphate allowed us to perform high confidence modeling with minimal assumptions of two reaction steps, the nucleophilic attack and a transition state. While L1-EN, APE1 and related nucleases are attractive therapeutic targets in theory, they represent poorly druggable structures. Significant efforts to identify inhibitors of APE1, a promising anticancer drug target, through biochemical high throughput and virtual ligand screenings were not successful; the most potent inhibitors had IC50s in the micromolar range (80–82). Compared to APE1, L1-EN has a shallower surface at the DNA binding interface and shorter loops surrounding the DNA binding site. These structural differences suggest that it will be even more difficult to find high affinity small molecule inhibitors of L1-EN than APE1. Additional studies of L1-EN and of the entire ORF2p are required to validate novel structure-based hypotheses, further define the mechanism of L1 retrotransposition, and identify strategies for inhibiting the process for therapeutic gain against human disease and aging.

## DATA AVAILABILITY

Structures determined in this study were deposited in the Worldwide Protein Data Bank (PDB) with accession numbers of PBD 7N8S for the L1-EN-D145A/Y226K/***dna14*** complex, PDB 7N94 for the L1-EN-D145A/Y226K/***dna17*** complex, and PDB 7N8K for Mg^2+^-bound wild type L1-EN.

## Supplementary Material

gkab826_Supplemental_Files

## References

[1] Goodier J.L. Restricting retrotransposons: a review. Mob DNA. 2016; 7:16. 27525044 10.1186/s13100-016-0070-z PMC4982230

[2] Kines K.J. , Sokolowski M., deHaro D.L., Christian C.M., Belancio V.P. Potential for genomic instability associated with retrotranspositionally-incompetent L1 loci. Nucleic Acids Res. 2014; 42:10488–10502. 25143528 10.1093/nar/gku687 PMC4176336

[3] Robberecht C. , Voet T., Zamani Esteki M., Nowakowska B.A., Vermeesch J.R. Nonallelic homologous recombination between retrotransposable elements is a driver of de novo unbalanced translocations. Genome Res. 2013; 23:411–418. 23212949 10.1101/gr.145631.112 PMC3589530

[4] Hohjoh H. , Singer M.F. Sequence-specific single-strand RNA binding protein encoded by the human LINE-1 retrotransposon. EMBO J. 1997; 16:6034–6043. 9312060 10.1093/emboj/16.19.6034 PMC1170233

[5] Dai L. , LaCava J., Taylor M.S., Boeke J.D. Expression and detection of LINE-1 ORF-encoded proteins. Mob Genet Elements. 2014; 4:e29319. 25054082 10.4161/mge.29319 PMC4091050

[6] Feng Q. , Moran J.V., Kazazian H.H. Jr, Boeke J.D. Human L1 retrotransposon encodes a conserved endonuclease required for retrotransposition. Cell. 1996; 87:905–916. 8945517 10.1016/s0092-8674(00)81997-2

[7] Cost G.J. , Boeke J.D. Targeting of human retrotransposon integration is directed by the specificity of the L1 endonuclease for regions of unusual DNA structure. Biochemistry. 1998; 37:18081–18093. 9922177 10.1021/bi981858s

[8] Hohjoh H. , Singer M.F. Cytoplasmic ribonucleoprotein complexes containing human LINE-1 protein and RNA. EMBO J. 1996; 15:630–639. 8599946 PMC449981

[9] Kulpa D.A. , Moran J.V. Cis-preferential LINE-1 reverse transcriptase activity in ribonucleoprotein particles. Nat. Struct. Mol. Biol. 2006; 13:655–660. 16783376 10.1038/nsmb1107

[10] Kazazian H.H. Jr , Wong C., Youssoufian H., Scott A.F., Phillips D.G., Antonarakis S.E Haemophilia A resulting from de novo insertion of L1 sequences represents a novel mechanism for mutation in man. Nature. 1988; 332:164–166. 2831458 10.1038/332164a0

[11] Moran J.V. , Holmes S.E., Naas T.P., DeBerardinis R.J., Boeke J.D., Kazazian H.H. Jr High frequency retrotransposition in cultured mammalian cells. Cell. 1996; 87:917–927. 8945518 10.1016/s0092-8674(00)81998-4

[12] Doucet A.J. , Hulme A.E., Sahinovic E., Kulpa D.A., Moldovan J.B., Kopera H.C., Athanikar J.N., Hasnaoui M., Bucheton A., Moran J.V. et al. Characterization of LINE-1 ribonucleoprotein particles. PLos Genet. 2010; 6:e1001150. 20949108 10.1371/journal.pgen.1001150 PMC2951350

[13] Martin S.L. , Bushman F.D. Nucleic acid chaperone activity of the ORF1 protein from the mouse LINE-1 retrotransposon. Mol. Cell. Biol. 2001; 21:467–475. 11134335 10.1128/MCB.21.2.467-475.2001 PMC86601

[14] Khazina E. , Weichenrieder O. Non-LTR retrotransposons encode noncanonical RRM domains in their first open reading frame. PNAS. 2009; 106:731–736. 19139409 10.1073/pnas.0809964106 PMC2630067

[15] Khazina E. , Truffault V., Buttner R., Schmidt S., Coles M., Weichenrieder O. Trimeric structure and flexibility of the L1ORF1 protein in human L1 retrotransposition. Nat. Struct. Mol. Biol. 2011; 18:1006–1014. 21822284 10.1038/nsmb.2097

[16] Khazina E. , Weichenrieder O. Human LINE-1 retrotransposition requires a metastable coiled coil and a positively charged N-terminus in L1ORF1p. eLife. 2018; 7:e34960. 29565245 10.7554/eLife.34960 PMC5940361

[17] Mathias S.L. , Scott A.F., Kazazian H.H. Jr, Boeke J.D., Gabriel A. Reverse transcriptase encoded by a human transposable element. Science. 1991; 254:1808–1810. 1722352 10.1126/science.1722352

[18] Clements A.P. , Singer M.F. The human LINE-1 reverse transcriptase:effect of deletions outside the common reverse transcriptase domain. Nucleic Acids Res. 1998; 26:3528–3535. 9671814 10.1093/nar/26.15.3528 PMC147723

[19] Piskareva O. , Ernst C., Higgins N., Schmatchenko V. The carboxy-terminal segment of the human LINE-1 ORF2 protein is involved in RNA binding. FEBS Open Bio. 2013; 3:433–437. 10.1016/j.fob.2013.09.005 PMC3821027 24251107

[20] Piskareva O. , Schmatchenko V. DNA polymerization by the reverse transcriptase of the human L1 retrotransposon on its own template in vitro. FEBS Lett. 2006; 580:661–668. 16412437 10.1016/j.febslet.2005.12.077

[21] Weichenrieder O. , Repanas K., Perrakis A. Crystal structure of the targeting endonuclease of the human LINE-1 retrotransposon. Structure. 2004; 12:975–986. 15274918 10.1016/j.str.2004.04.011

[22] Luan D.D. , Korman M.H., Jakubczak J.L., Eickbush T.H. Reverse transcription of R2Bm RNA is primed by a nick at the chromosomal target site: a mechanism for non-LTR retrotransposition. Cell. 1993; 72:595–605. 7679954 10.1016/0092-8674(93)90078-5

[23] Cost G.J. , Feng Q., Jacquier A., Boeke J.D. Human L1 element target-primed reverse transcription in vitro. EMBO J. 2002; 21:5899–5910. 12411507 10.1093/emboj/cdf592 PMC131089

[24] Repanas K. , Zingler N., Layer L.E., Schumann G.G., Perrakis A., Weichenrieder O. Determinants for DNA target structure selectivity of the human LINE-1 retrotransposon endonuclease. Nucleic Acids Res. 2007; 35:4914–4926. 17626046 10.1093/nar/gkm516 PMC1950540

[25] Jurka J. Sequence patterns indicate an enzymatic involvement in integration of mammalian retroposons. PNAS. 1997; 94:1872–1877. 9050872 10.1073/pnas.94.5.1872 PMC20010

[26] Viollet S. , Monot C., Cristofari G. L1 retrotransposition: The snap-velcro model and its consequences. Mob Genet Elements. 2014; 4:e28907. 24818067 10.4161/mge.28907 PMC4014453

[27] Monot C. , Kuciak M., Viollet S., Mir A.A., Gabus C., Darlix J.L., Cristofari G. The specificity and flexibility of l1 reverse transcription priming at imperfect T-tracts. PLos Genet. 2013; 9:e1003499. 23675310 10.1371/journal.pgen.1003499 PMC3649969

[28] Zhang X. , Zhang R., Yu J. New understanding of the relevant role of LINE-1 retrotransposition in human disease and immune modulation. Front. Cell Dev. Biol. 2020; 8:657. 32850797 10.3389/fcell.2020.00657 PMC7426637

[29] Burns K.H. Transposable elements in cancer. Nat. Rev. Cancer. 2017; 17:415–424. 28642606 10.1038/nrc.2017.35

[30] Anwar S.L. , Wulaningsih W., Lehmann U. Transposable elements in human cancer: causes and consequences of deregulation. Int. J. Mol. Sci. 2017; 18:974. 10.3390/ijms18050974 PMC5454887 28471386

[31] Song Y.S. , Kim Y., Cho N.Y., Yang H.K., Kim W.H., Kang G.H. Methylation status of long interspersed element-1 in advanced gastric cancer and its prognostic implication. Gastric Cancer. 2016; 19:98–106. 25609453 10.1007/s10120-015-0463-6

[32] Rodriguez-Martin B. , Alvarez E.G., Baez-Ortega A., Zamora J., Supek F., Demeulemeester J., Santamarina M., Ju Y.S., Temes J., Garcia-Souto D. et al. Pan-cancer analysis of whole genomes identifies driver rearrangements promoted by LINE-1 retrotransposition. Nat. Genet. 2020; 52:306–319. 32024998 10.1038/s41588-019-0562-0 PMC7058536

[33] Burns K.H. Our conflict with transposable elements and its implications for human disease. Annu Rev Pathol. 2020; 15:51–70. 31977294 10.1146/annurev-pathmechdis-012419-032633

[34] Pereira G.C. , Sanchez L., Schaughency P.M., Rubio-Roldan A., Choi J.A., Planet E., Batra R., Turelli P., Trono D., Ostrow L.W. et al. Properties of LINE-1 proteins and repeat element expression in the context of amyotrophic lateral sclerosis. Mob DNA. 2018; 9:35. 30564290 10.1186/s13100-018-0138-z PMC6295051

[35] Liu E.Y. , Russ J., Cali C.P., Phan J.M., Amlie-Wolf A., Lee E.B. Loss of nuclear TDP-43 is associated with decondensation of LINE retrotransposons. Cell Rep. 2019; 27:1409–1421. 31042469 10.1016/j.celrep.2019.04.003 PMC6508629

[36] Baeken M.W. , Moosmann B., Hajieva P. Retrotransposon activation by distressed mitochondria in neurons. Biochem. Biophys. Res. Commun. 2020; 525:570–575. 32115149 10.1016/j.bbrc.2020.02.106

[37] Gamdzyk M. , Doycheva D.M., Araujo C., Ocak U., Luo Y., Tang J., Zhang J.H. cGAS/STING pathway activation contributes to delayed neurodegeneration in neonatal hypoxia-ischemia rat model: possible involvement of LINE-1. Mol. Neurobiol. 2020; 57:2600–2619. 32253733 10.1007/s12035-020-01904-7 PMC7260114

[38] Savage A.L. , Lopez A.I., Iacoangeli A., Bubb V.J., Smith B., Troakes C., Alahmady N., Koks S., Schumann G.G., Al-Chalabi A. et al. Frequency and methylation status of selected retrotransposition competent L1 loci in amyotrophic lateral sclerosis. Mol Brain. 2020; 13:154. 33187550 10.1186/s13041-020-00694-2 PMC7666467

[39] Pfaff A.L. , Bubb V.J., Quinn J.P., Koks S. An increased burden of highly active retrotransposition competent L1s is associated with Parkinson's disease risk and progression in the PPMI cohort. Int. J. Mol. Sci. 2020; 21:6562. 10.3390/ijms21186562 PMC7554759 32911699

[40] Simon M. , Van Meter M., Ablaeva J., Ke Z., Gonzalez R.S., Taguchi T., De Cecco M., Leonova K.I., Kogan V., Helfand S.L. et al. LINE1 derepression in aged wild-type and SIRT6-deficient mice drives inflammation. Cell Metab. 2019; 29:871–885. 30853213 10.1016/j.cmet.2019.02.014 PMC6449196

[41] De Cecco M. , Ito T., Petrashen A.P., Elias A.E., Skvir N.J., Criscione S.W., Caligiana A., Brocculi G., Adney E.M., Boeke J.D. et al. L1 drives IFN in senescent cells and promotes age-associated inflammation. Nature. 2019; 566:73–78. 30728521 10.1038/s41586-018-0784-9 PMC6519963

[42] De Cecco M. , Ito T., Petrashen A.P., Elias A.E., Skvir N.J., Criscione S.W., Caligiana A., Brocculi G., Adney E.M., Boeke J.D. et al. L1 drives IFN in senescent cells and promotes age-associated inflammation. Nature. 2019; 572:E5. 31296937 10.1038/s41586-019-1350-9 PMC7017651

[43] Rozek L.S. , Virani S., Bellile E.L., Taylor J.M.G., Sartor M.A., Zarins K.R., Virani A., Cote C., Worden F.P., Mark M.E.P. et al. Soy isoflavone supplementation increases long interspersed nucleotide element-1 (LINE-1) methylation in head and neck squamous cell carcinoma. Nutr. Cancer. 2019; 71:772–780. 30862188 10.1080/01635581.2019.1577981 PMC6513708

[44] Wang X.Y. , Zhang Y., Yang N., Cheng H., Sun Y.J. DNMT3a mediates paclitaxel-induced abnormal expression of LINE-1 by increasing the intragenic methylation. Yi Chuan. 2020; 42:100–111. 31956100 10.16288/j.yczz.19-258

[45] Guler G.D. , Tindell C.A., Pitti R., Wilson C., Nichols K., KaiWai Cheung T., Kim H.J., Wongchenko M., Yan Y., Haley B. et al. Repression of stress-induced LINE-1 expression protects cancer cell subpopulations from lethal drug exposure. Cancer Cell. 2017; 32:221–237. 28781121 10.1016/j.ccell.2017.07.002

[46] Sciamanna I. , Sinibaldi-Vallebona P., Serafino A., Spadafora C. LINE-1-encoded reverse Transcriptase as a target in cancer therapy. Front. Biosci. (Landmark Ed.). 2018; 23:1360–1369. 29293438 10.2741/4648

[47] Dlakic M. Functionally unrelated signalling proteins contain a fold similar to Mg2+-dependent endonucleases. Trends Biochem. Sci. 2000; 25:272–273. 10838565 10.1016/s0968-0004(00)01582-6

[48] Lu S. , Wang J., Chitsaz F., Derbyshire M.K., Geer R.C., Gonzales N.R., Gwadz M., Hurwitz D.I., Marchler G.H., Song J.S. et al. CDD/SPARCLE: the conserved domain database in 2020. Nucleic Acids Res. 2020; 48:D265–D268. 31777944 10.1093/nar/gkz991 PMC6943070

[49] Zingler N. , Weichenrieder O., Schumann G.G. APE-type non-LTR retrotransposons: determinants involved in target site recognition. Cytogenet. Genome Res. 2005; 110:250–268. 16093679 10.1159/000084959

[50] Gilbert N. , Lutz S., Morrish T.A., Moran J.V. Multiple fates of L1 retrotransposition intermediates in cultured human cells. Mol. Cell. Biol. 2005; 25:7780–7795. 16107723 10.1128/MCB.25.17.7780-7795.2005 PMC1190285

[51] Flasch D.A. , Macia A., Sanchez L., Ljungman M., Heras S.R., Garcia-Perez J.L., Wilson T.E., Moran J.V. Genome-wide de novo L1 retrotransposition connects endonuclease activity with replication. Cell. 2019; 177:837–851. 30955886 10.1016/j.cell.2019.02.050 PMC6558663

[52] Martin F. , Maranon C., Olivares M., Alonso C., Lopez M.C. Characterization of a non-long terminal repeat retrotransposon cDNA (L1Tc) from Trypanosoma cruzi: homology of the first ORF with the ape family of DNA repair enzymes. J. Mol. Biol. 1995; 247:49–59. 7534829 10.1006/jmbi.1994.0121

[53] Repanas K. , Fuentes G., Cohen S.X., Bonvin A.M., Perrakis A. Insights into the DNA cleavage mechanism of human LINE-1 retrotransposon endonuclease. Proteins. 2009; 74:917–928. 18767160 10.1002/prot.22201

[54] El Hassan M.A. , Calladine C.R. Two distinct modes of protein-induced bending in DNA. J. Mol. Biol. 1998; 282:331–343. 9735291 10.1006/jmbi.1998.1994

[55] Stefl R. , Wu H., Ravindranathan S., Sklenar V., Feigon J. DNA A-tract bending in three dimensions: solving the dA4T4 vs. dT4A4 conundrum. PNAS. 2004; 101:1177–1182. 14739342 10.1073/pnas.0308143100 PMC337026

[56] Schuck P. Size-distribution analysis of macromolecules by sedimentation velocity ultracentrifugation and lamm equation modeling. Biophys. J. 2000; 78:1606–1619. 10692345 10.1016/S0006-3495(00)76713-0 PMC1300758

[57] Adams P.D. , Afonine P.V., Bunkoczi G., Chen V.B., Davis I.W., Echols N., Headd J.J., Hung L.W., Kapral G.J., Grosse-Kunstleve R.W. et al. PHENIX: a comprehensive Python-based system for macromolecular structure solution. Acta Crystallogr. D. Biol. Crystallogr. 2010; 66:213–221. 20124702 10.1107/S0907444909052925 PMC2815670

[58] Liebschner D. , Afonine P.V., Baker M.L., Bunkoczi G., Chen V.B., Croll T.I., Hintze B., Hung L.W., Jain S., McCoy A.J. et al. Macromolecular structure determination using X-rays, neutrons and electrons: recent developments in Phenix. Acta Crystallogr D Struct Biol. 2019; 75:861–877. 31588918 10.1107/S2059798319011471 PMC6778852

[59] Emsley P. , Lohkamp B., Scott W.G., Cowtan K. Features and development of Coot. Acta Crystallogr. D. Biol. Crystallogr. 2010; 66:486–501. 20383002 10.1107/S0907444910007493 PMC2852313

[60] Sagendorf J.M. , Berman H.M., Rohs R. DNAproDB: an interactive tool for structural analysis of DNA-protein complexes. Nucleic Acids Res. 2017; 45:W89–W97. 28431131 10.1093/nar/gkx272 PMC5570235

[61] Lu X.J. , Olson W.K. 3DNA: a versatile, integrated software system for the analysis, rebuilding and visualization of three-dimensional nucleic-acid structures. Nat. Protoc. 2008; 3:1213–1227. 18600227 10.1038/nprot.2008.104 PMC3065354

[62] Nguyen S.P. , Li Z., Xu D., Shang Y. New Deep Learning Methods for Protein Loop Modeling. IEEE/ACM Trans. Comput. Biol. Bioinform. 2019; 16:596–606. 29990046 10.1109/TCBB.2017.2784434 PMC6580050

[63] Grazulis S. , Daskevic A., Merkys A., Chateigner D., Lutterotti L., Quiros M., Serebryanaya N.R., Moeck P., Downs R.T., Le Bail A. Crystallography Open Database (COD): an open-access collection of crystal structures and platform for world-wide collaboration. Nucleic Acids Res. 2012; 40:D420–D427. 22070882 10.1093/nar/gkr900 PMC3245043

[64] Timosheva N.V. , Chandrasekaran A., Holmes R.R. Biologically relevant phosphoranes: structural characterization of glucofuranose- and xylofuranose-based phosphoranes. Inorg. Chem. 2006; 45:3113–3123. 16562968 10.1021/ic052105e

[65] Lahm A. , Suck D. DNase I-induced DNA conformation. 2 A structure of a DNase I-octamer complex. J. Mol. Biol. 1991; 222:645–667. 1748997 10.1016/0022-2836(91)90502-w

[66] Mol C.D. , Izumi T., Mitra S., Tainer J.A. DNA-bound structures and mutants reveal abasic DNA binding by APE1 and DNA repair coordination [corrected]. Nature. 2000; 403:451–456. 10667800 10.1038/35000249

[67] Freudenthal B.D. , Beard W.A., Cuneo M.J., Dyrkheeva N.S., Wilson S.H. Capturing snapshots of APE1 processing DNA damage. Nat. Struct. Mol. Biol. 2015; 22:924–931. 26458045 10.1038/nsmb.3105 PMC4654669

[68] Weston S.A. , Lahm A., Suck D. X-ray structure of the DNase I-d(GGTATACC)2 complex at 2.3 A resolution. J. Mol. Biol. 1992; 226:1237–1256. 1518054 10.1016/0022-2836(92)91064-v

[69] Yang W. Nucleases: diversity of structure, function and mechanism. Q. Rev. Biophys. 2011; 44:1–93. 20854710 10.1017/S0033583510000181 PMC6320257

[70] Herrera J.E. , Chaires J.B. Characterization of preferred deoxyribonuclease I cleavage sites. J. Mol. Biol. 1994; 236:405–411. 8107130 10.1006/jmbi.1994.1152

[71] Svozil D. , Kalina J., Omelka M., Schneider B. DNA conformations and their sequence preferences. Nucleic Acids Res. 2008; 36:3690–3706. 18477633 10.1093/nar/gkn260 PMC2441783

[72] Vlieghe D. , Turkenburg J.P., Van Meervelt L. B-DNA at atomic resolution reveals extended hydration patterns. Acta Crystallogr. D. Biol. Crystallogr. 1999; 55:1495–1502. 10489444 10.1107/s0907444999007933

[73] Varnai P. , Djuranovic D., Lavery R., Hartmann B. Alpha/gamma transitions in the B-DNA backbone. Nucleic Acids Res. 2002; 30:5398–5406. 12490708 10.1093/nar/gkf680 PMC140057

[74] Parsiegla G. , Noguere C., Santell L., Lazarus R.A., Bourne Y. The structure of human DNase I bound to magnesium and phosphate ions points to a catalytic mechanism common to members of the DNase I-like superfamily. Biochemistry. 2012; 51:10250–10258. 23215638 10.1021/bi300873f

[75] He H. , Chen Q., Georgiadis M.M. High-resolution crystal structures reveal plasticity in the metal binding site of apurinic/apyrimidinic endonuclease I. Biochemistry. 2014; 53:6520–6529. 25251148 10.1021/bi500676p PMC4204877

[76] Beernink P.T. , Segelke B.W., Hadi M.Z., Erzberger J.P., Wilson D.M. 3rd, Rupp B. Two divalent metal ions in the active site of a new crystal form of human apurinic/apyrimidinic endonuclease, Ape1: implications for the catalytic mechanism. J. Mol. Biol. 2001; 307:1023–1034. 11286553 10.1006/jmbi.2001.4529

[77] Ikeda T. , Boero M., Terakura K. Hydration properties of magnesium and calcium ions from constrained first principles molecular dynamics. J. Chem. Phys. 2007; 127:074503. 17718616 10.1063/1.2768063

[78] Olsson M.H. , Sondergaard C.R., Rostkowski M., Jensen J.H. PROPKA3: consistent treatment of internal and surface residues in empirical pKa predictions. J. Chem. Theory Comput. 2011; 7:525–537. 26596171 10.1021/ct100578z

[79] Tsutakawa S.E. , Shin D.S., Mol C.D., Izumi T., Arvai A.S., Mantha A.K., Szczesny B., Ivanov I.N., Hosfield D.J., Maiti B. et al. Conserved structural chemistry for incision activity in structurally non-homologous apurinic/apyrimidinic endonuclease APE1 and endonuclease IV DNA repair enzymes. J. Biol. Chem. 2013; 288:8445–8455. 23355472 10.1074/jbc.M112.422774 PMC3605660

[80] Rai G. , Vyjayanti V.N., Dorjsuren D., Simeonov A., Jadhav A., Wilson D.M. 3rd, Maloney D.J. Synthesis, biological evaluation, and structure-activity relationships of a novel class of apurinic/apyrimidinic endonuclease 1 inhibitors. J. Med. Chem. 2012; 55:3101–3112. 22455312 10.1021/jm201537d PMC3515842

[81] Mohammed M.Z. , Vyjayanti V.N., Laughton C.A., Dekker L.V., Fischer P.M., Wilson D.M. 3rd, Abbotts R., Shah S., Patel P.M., Hickson I.D. et al. Development and evaluation of human AP endonuclease inhibitors in melanoma and glioma cell lines. Br. J. Cancer. 2011; 104:653–663. 21266972 10.1038/sj.bjc.6606058 PMC3049581

[82] Wilson D.M. 3rd , Simeonov A. Small molecule inhibitors of DNA repair nuclease activities of APE1. Cell. Mol. Life Sci. 2010; 67:3621–3631. 20809131 10.1007/s00018-010-0488-2 PMC2956791

