# Supplementary material for: Structural dissection of sequence recognition and catalytic mechanism of human LINE-1 endonuclease
Source: Nucleic Acids Res. 2021 Sep 23;49(19):11350–66. doi: 10.1093/nar/gkab826 (PMC8565326; doi:10.1093/nar/gkab826)
Supplement: gkab826_Supplemental_Files [file gkab826_supplemental_files.zip › Supplementary materials rev1.pdf]

Structural dissection of sequence recognition and catalytic mechanism of human LINE1 endonuclease

Ian Miller[1], Max Totrov[2], Lioubov Korotchkina[3], Denis Kazulkin[3], Andrei Gudkov[3,4], and Sergey Korolev[1]*

Supplementary Materials

Supplementary Table 1

Enzymatic activities of wild type (WT) L1-EN, L1-EN/D145A and L1-EN/Y226K determined in fluorescent probe assay.

**L1-EN**	**In vitro endonuclease activity (RFU/min/ng, mean ±SD)**	**% endonuclease activity relative to WT**
WT	0.446 ±0.111 (n=5)	100 ±25
D145A	0.011 ±0.001 (n=5)	2.4 ±0.2
Y226K	0.992 ±0.273 (n=4)	222 ±70

Supplementary Table 2

Main chain torsion angles of canonical B-form, of two alternative conformations in dna14 and of conformation in crystal structure 431D.

Angle	Atoms	B-DNA	***dna14***, A11	***dna14***, A11 rotated	431D base 13
α	O3′$_{-1}$-P-O5′-C5′	**-61**	**-56.**	**50.**	**63.**
β	P-O5′-C5′-C4′	**179.**	**155.**	**-137.**	**-145.**
γ	O5′-C5′-C4′-C3′	**48.**	**49.**	**-155.**	**-173.**
δ	C5′-C4′-C3′-O3′	132.	139.	145.	85.
ε	C4′-C3′-O3′-P$_{+1}$	-178.	-177.	-171.	-158.
ζ	C3′-O3′-P$_{+1}$-O5′$_{+1}$	-97.	-98.5	-97.	-69.

Supplementary Figure 1

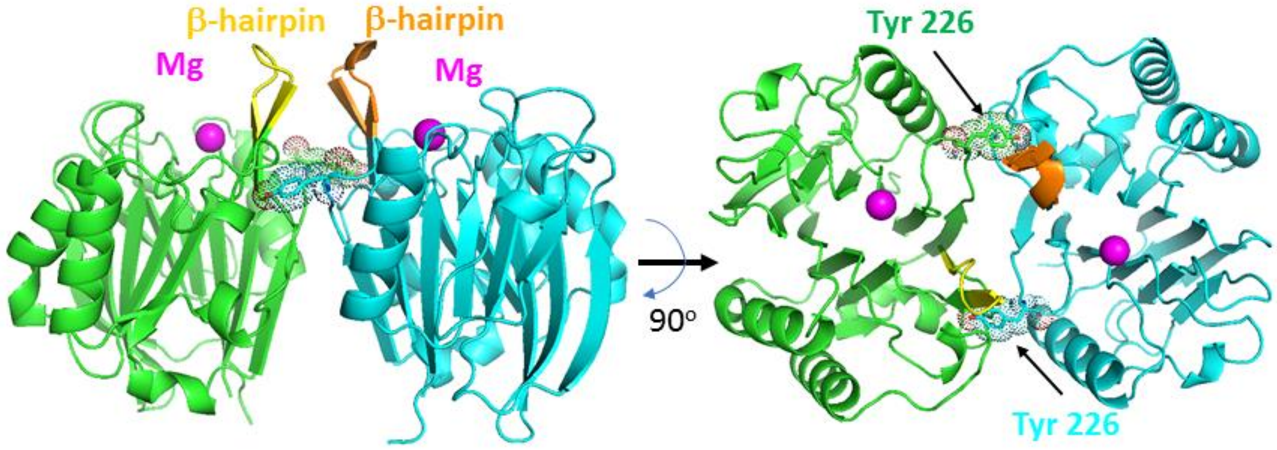

Crystallographic dimers observed in published structures of L1-EN. Orthogonal views of L1-EN dimer observed in crystal structure of L1-EN with Mg2++ formed by symmetry-related monomers shown in cartoon representation in green and blue. Minor groove binding β-hairpins are colored by yellow and orange. Active sites are depicted by Mg2+ ion shown by magenta sphere. Tyr 226 mutated for co-crystallization with DNA are shown in sticks and dots representation.

Supplementary Figure 2

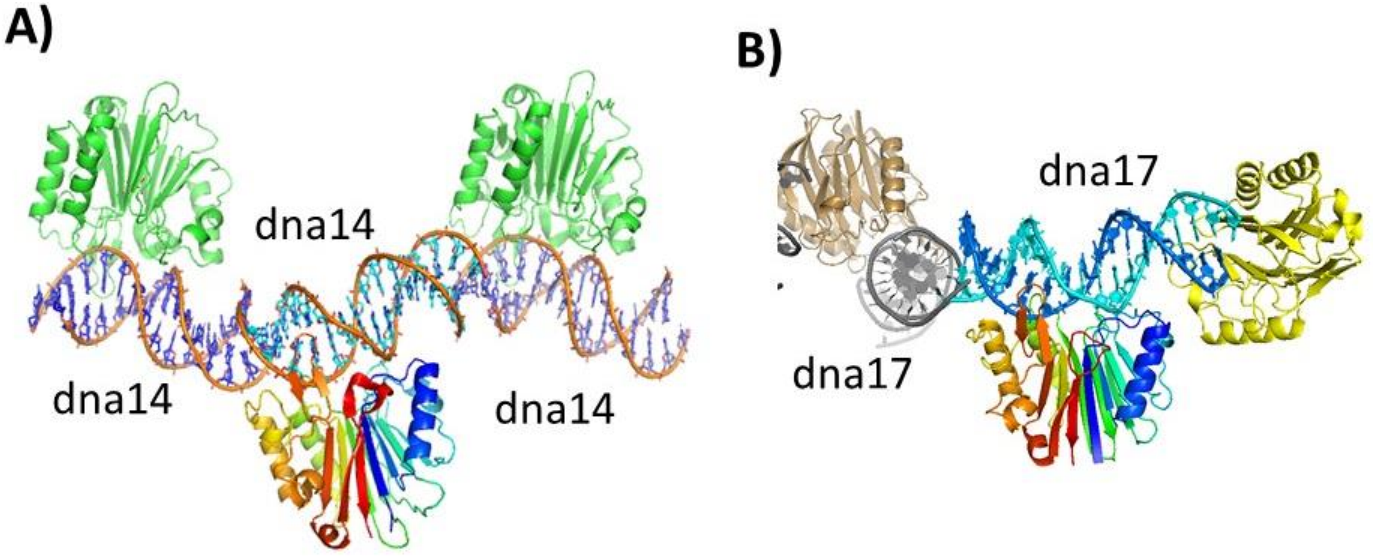

Crystal packing of L1-DNA complexes*. A)** Three L1-EN-bound complexes formed by three ***dna14 substrates are shown. The DNA duplexes interact through the single nucleotide A/T overhangs at their ends, which form Watson-Crick base pairs resulting in a continuous B-form DNA helix. **B)** Interactions of ***dna17*** substrate (blue and cyan) with symmetry-related molecules including a symmetry related L1-EN on one side and a backbone of the symmetry-related DNA substrate on the other side. Both interactions disrupt Watson-Crick base pairing.

Supplementary Figure 3

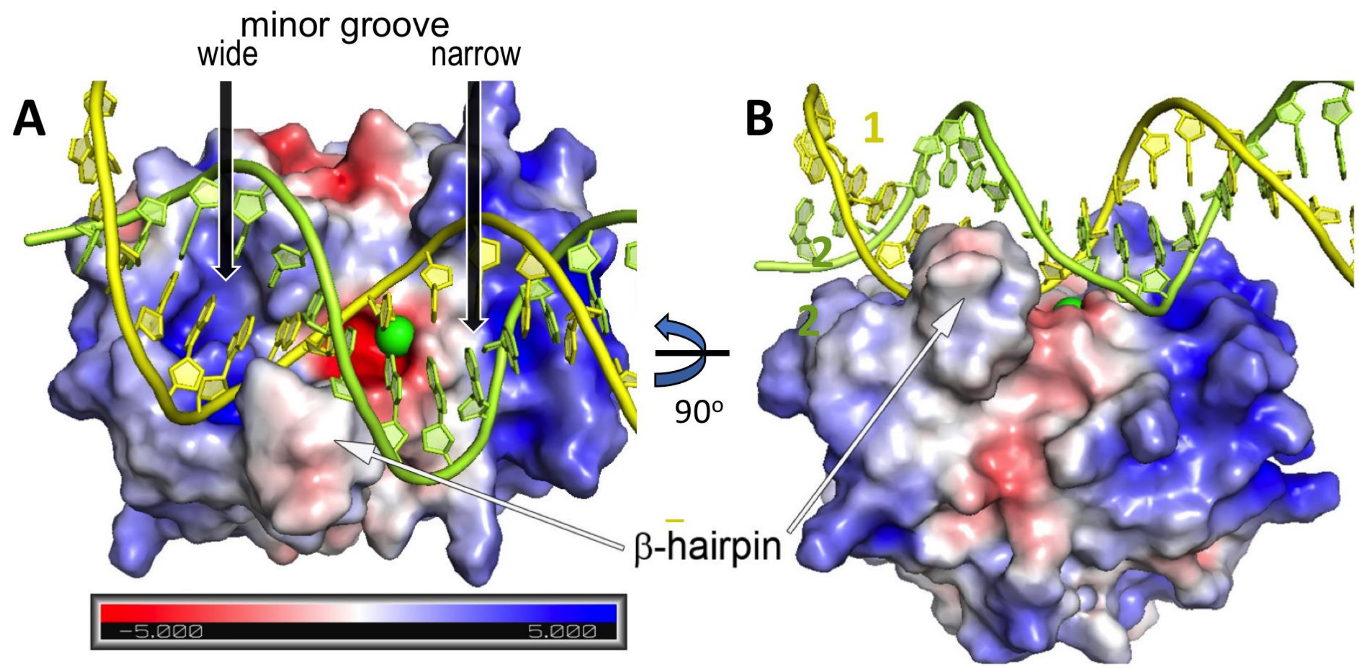

Electrostatic potential surface representation of L1-EN with bound DNA. **A)** Top and **B)** side views of the surface representation of L1-EN-D145A/Y226K color-coded according to electrostatic potential with bound DNA shown in cartoon representation. Electrostatic potential is indicated by the color scale shown below (**A**), with the intensity of red and blue color corresponding to negative and positive charge, respectively. The Mg^{2+} ion at the active site is shown as a green sphere. Areas of the minor groove that are wider or narrower than in B-form DNA are pointed out by black arrows. DNA strand 1 (the nicked strand) is shown in yellow and strand 2 is shown in green. The βB5-βB6 β-hairpin loop is indicated by the white arrows.

Supplementary Figure 4

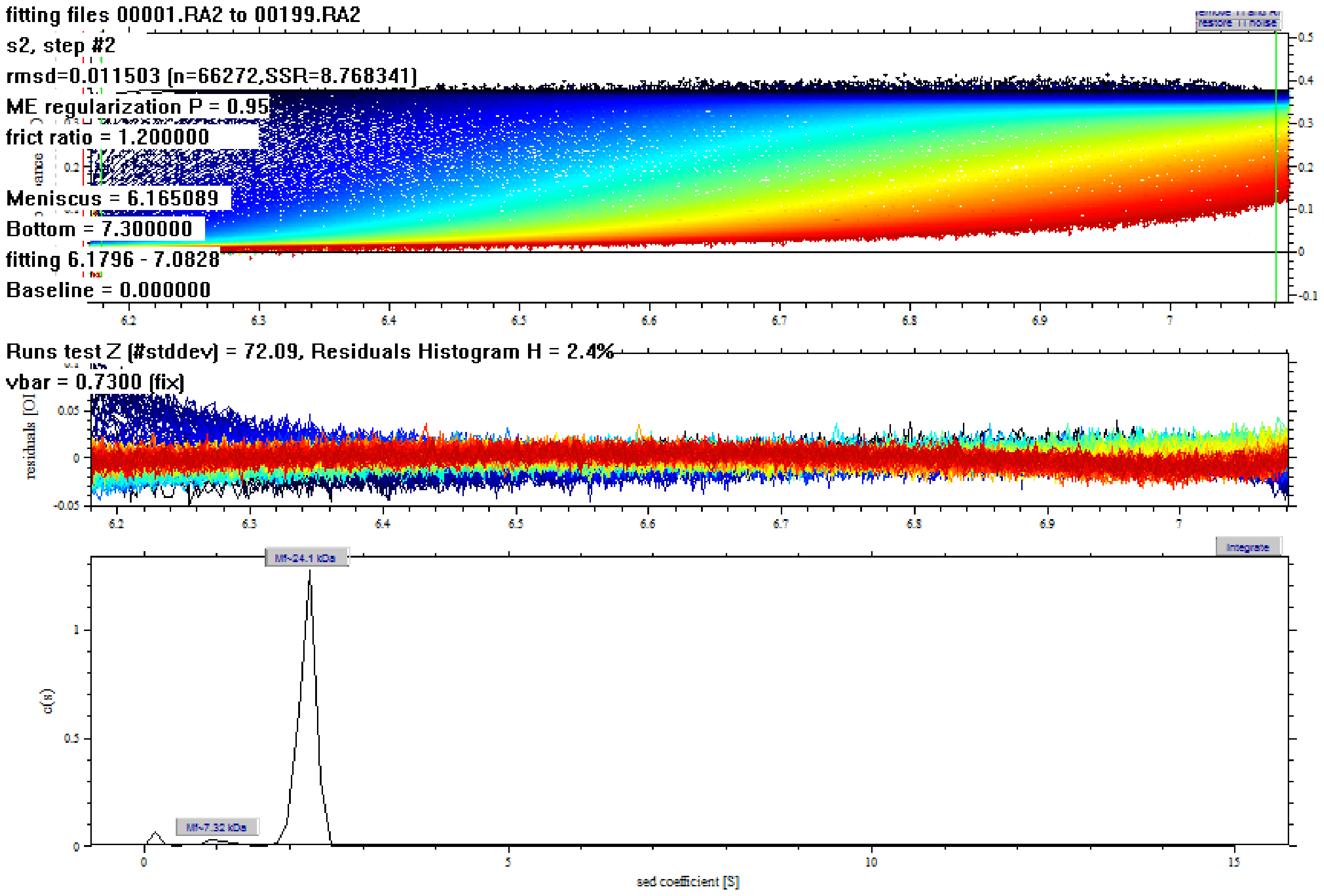

Sedimentation velocity results for wild type L1-EN. The observed sedimentation coefficient peak with estimated MW of 24 kDa indicates a monomeric form of the protein (MW = 27 kDa).

Supplementary Movie 1.

Modeling of the catalytic mechanism of L1-EN. Nucleophilic activation of the scissile bond phosphate and formation of the transition state during phosphate rotation observed in crystal structure.
